# Cathelicidin-related antimicrobial peptide protects against enteric pathogen-accelerated type 1 diabetes in mice

**DOI:** 10.7150/thno.61433

**Published:** 2022-04-24

**Authors:** Lingling Jia, Jiahong Li, Ming Zhang, He Liu, Zhengnan Ren, Xiao Liang Dong, Xiaohua Pan, Ju Qiu, Li-Long Pan, Jia Sun

**Affiliations:** 1 Wuxi School of Medicine and School of Food Science and Technology, Jiangnan University, Wuxi 214122, Jiangsu, P. R. China.; 2 State Key Laboratory of Food Science and Technology, Jiangnan University, Wuxi 214122, Jiangsu, P. R. China.; 3 College of Food and Pharmaceutical Sciences, Ningbo University, Ningbo 315800, Zhejiang, P. R. China.; 4 CAS Key Laboratory of Tissue Microenvironment and Tumor, Shanghai Institute of Nutrition and Health, Shanghai Institutes for Biological Sciences, University of Chinese Academy of Sciences, Chinese Academy of Sciences, Shanghai 200031, P. R. China.

**Keywords:** antimicrobial peptides, gut barrier, type 1 diabetes, gut-pancreas crosstalk, intestinal IFNγ^+^ T cell migration

## Abstract

**Rationale:** Gut barrier disruption caused by enteric pathogen infection results in activated diabetogenic T cells and accelerated type 1 diabetes (T1D). Cathelicidin-related antimicrobial peptide (CRAMP) maintains intestinal barrier integrity, regulates the microbiome, and exerts positive immune-modulatory effects on pancreatic diseases.

**Methods:** The model enteric pathogen *Citrobacter rodentium (C. rodentium)* was adopted to represent clinical colonic infection with gut barrier disruption. The protective role and gut-pancreas pathophysiological mechanism of CRAMP in enteric pathogen-accelerated T1D were investigated in spontaneous non-obese diabetic (NOD) mice and streptozotocin-induced diabetic mice.

**Results:** Colonic CRAMP production was defective in *C. rodentium* infection-accelerated T1D. *C. rodentium* infection triggered the recruitment of interferon-gamma (IFN-γ)^+^ T cells and accelerated T1D. In the *C. rodentium*-accelerated T1D mice, CRAMP deficiency further aggravated gut barrier disruption, gut dysbiosis, and diabetic phenotype, which could be reversed by CRAMP treatment. The protective effect of CRAMP may be due to CRAMP inhibiting *C. rodentium*-aggravated gut immune dysregulation, gut dysbiosis, and migration of gut-primed IFN-γ^+^ T cells to the pancreas, thus contributing to gut barrier protection and pancreatic-intestinal immune homeostasis.

**Conclusion:** CRAMP plays a pivotal role in pancreatic-gut crosstalk during *C. rodentium*-accelerated T1D by gut barrier-protective, immune- and microbial-modulatory mechanisms. Cathelicidin supplementation to restore a healthy gut barrier may represent a novel therapeutic strategy for T1D.

## Introduction

Type 1 diabetes (T1D) is a chronic autoimmune disease, of which the triggering and intermediary mechanisms remain to be fully understood 1. The global incidence of T1D continues on the rise, with the acceleration rate far outpacing the genetic variation rate, suggesting that environmental factors play a significant role in its onset 1. The gastrointestinal barrier is a fundamental gatekeeper to avoid contact between luminal content and the human body. Disruption of the intestinal barrier promotes the initiation and development of various autoimmune diseases, including T1D 2, 3. More interestingly, functional loss of intestinal barrier integrity and the occurrence of low-grade intestinal inflammation precede the onset of T1D in patients and preclinical models 2. Gut infections are epidemiologically associated with T1D 4-6. Moreover, intestinal infection, with resultant intestinal barrier disruption, accelerates the progression of T1D 4-6. However, the mechanisms involved in the pancreatic-intestinal crosstalk remain unknown. Gut barrier disruption and consequent escape of microbial products may be one of the environmental triggers for T1D 7. Thus, in diabetes-prone individuals, the inhibition of gut barrier disruption may represent an effective therapeutic strategy for T1D.

Antimicrobial peptides (AMPs) are evolutionarily conserved molecules found in organisms ranging from prokaryotes to humans as an essential host defense mechanism against invading microbes 8, 9. Apart from their antimicrobial activities, additional immune-modulatory functions of AMPs have been increasingly appreciated 10. The cathelicidin family of AMPs (named LL-37 in humans and cathelicidin-related AMP [CRAMP] in mice) are primarily present at the host colon-microbe interface, and their effects have been largely described for intestinal inflammatory diseases or antimicrobial activities against various gut microbes 11-14. More dramatically, recent studies have indicated that CRAMP in both colon and pancreas maintains intestinal barrier integrity, shapes the microbiome, and dampens the development of pancreatic diseases, including T1D 15, 16. However, whether and how CRAMP plays a role in pancreatic-gut crosstalk remains poorly understood.

In this study, we applied the model enteric pathogen *Citrobacter rodentium (C. rodentium)* to induce gut infection with intestinal barrier disruption to mimic clinical enteric pathogen infection-induced T1D 4, 11, 17-20. The role and gut-pancreas pathophysiological mechanisms of CRAMP in *C. rodentium*-accelerated T1D were investigated in spontaneous non-obese diabetic (NOD) mice and streptozotocin (STZ)-induced diabetic CRAMP deficient *Cnlp^-/-^* mice. This study will shed light on the cellular-, immune- and microbial-modulatory mechanisms underlying gut-pancreatic crosstalk to facilitate the development of AMPs-based therapy for T1D.

## Materials and Methods

### Mice

Three-week-old male *Cnlp*^-/-^ (the gene encodes CRAMP) mice (Jackson Laboratory, CA, China) 11, male C57BL/6J wide-type (WT) mice (Su Pu Si Biotechnology, Jiangsu, China), and female NOD (Su Pu Si Biotechnology) mice (weighing 13-16 g) were housed under a specific pathogen-free environment at the animals housing unit of Jiangnan University (Jiangsu, China). All mice were housed in individual ventilated caging systems (Tecniplast, Rome, Italy) with controlled temperature (24 ± 1 °C) and 12 h light-dark cycle and had free access to water and standard chow. All studies were approved by the Institutional Animal Ethics Committee of Jiangnan University (JN. No. 20180415c3131220 [67]) and carried out in compliance with national and international guidelines for the Care and Use of Laboratory Animals.

### *C. rodentium* infection and induction of diabetes by multiple low-dose streptozotocin

The model enteric pathogen *C. rodentium* was used to induce gut infection with intestinal barrier disruption to mimic the enteric pathogen infection in humans and pathogen infection-induced T1D 4, 11, 17-20. *C. rodentium* strain DBS100 (ATCC 51459; American Type Culture Collection, CA, USA) was prepared by culturing in LB broth overnight, and bacterial concentration was determined by measuring the optical density at 600 nm. Three-week-old female NOD mice were infected with *C. rodentium* strain (2.5×10^10^ CFU in 200 μL LB broth) by gavage along with the corresponding intervention (n = 15), as previously described 4, 21. Fecal samples were collected, weighed, and then homogenized in sterile PBS. Serially diluted homogenates were plated on MacConkey agar plates. The colonies were identified based on morphology after 24 h of incubation at 37 °C 21. Three-week-old WT and* Cnlp*^-/-^ mice (n = 12) were infected with the *C. rodentium* weekly along with the corresponding intervention as previously described 4, 21. Infected mice were then injected with low-dose (45 mg·kg^-1^ a day) of STZ (Sigma S0130, St. Louis, MO, USA) by intraperitoneal injection for 5 consecutive days to induce T1D 15. The control mice received only sodium citrate buffer (vehicle) i.p. for five consecutive days. All the mice received CRAMP (100 μg, the mature form; 088328; GL biochem, Shanghai, China) or saline by intraperitoneal injection or intracolonic administration twice a week 15. The short schematics for each treatment are shown in **Figure S1**.

NOD mice were randomly assigned to three groups: (1) NOD mice: the NOD control mice; (2) NOD-*C.r* mice: the *C. rodentium-*infected NOD mice; (3) NOD-*C.r*-CRAMP mice: the* C. rodentium-*infected NOD mice treated with CRAMP.

The WT and* Cnlp*^-/-^ mice were randomly assigned to six groups: (1) WT mice: WT control mice; (2) WT-STZ mice: the WT mice treated with STZ; (3) WT-STZ-*C.r* mice: the *C. rodentium-*infected WT mice treated with STZ; (4) *Cnlp*^-/-^ mice: the *Cnlp^-/-^* control mice; (5) *Cnlp*^-/-^-STZ-*C.r* mice: the *C. rodentium-*infected *Cnlp^-/-^* mice treated with STZ; (6) *Cnlp*^-/-^-STZ-*C.r*-CRAMP mice: the *C. rodentium-*infected *Cnlp^-/-^* mice treated with STZ and CRAMP.

### Intracolonic administration

After fasting overnight, the mouse was anesthetized by isoflurane with an anesthesia machine air pump (R510-29, Rayward Life Technologies Inc, Shenzhen, China). The posterior end of the mouse was gently pressed to remove the feces that might be present in the distal colon. A 19 G needle with a fine bore polythene tubing (PE-50, 0.58 mm ID, 0.96 mm OD, PORTEX, Smiths Medical, Minneapolis, USA) fastened to its end was attached to a 1 ml syringe. The fine bore polythene tubing was gently inserted intrarectally, reaching approximately 3-4 cm proximal to the anus 22, 23. 100 μg of CRAMP was slowly injected, and the mouse was positioned head-down for 90 s to avoid CRAMP loss.

### Blood glucose measurement

A glucometer (Roche, NSW, USA) was used to measure the glucose from the tail vein after 6 h fasting, expressed in mmol·L^-1^. Mice were considered diabetic when glucose level > 11.1 mmol·L^-1^ after two consecutive determinations 7. Mice were considered to have severe diabetes when glucose level > 16.6 mmol·L^-1^ after two consecutive determinations 24.

The magnitude of the glucose response was represented by the total glucose area under the curve (AUC glucose), which was calculated using the trapezoidal rule.

### Oral glucose tolerance test

The mice received glucose by gavage (2 g·kg^-1^ body weight) after an 18 h fasting at 3 weeks after STZ injection, and blood glucose was measured at 0, 15, 30, 60, and 120 min.

### Samples collection

Mice were sacrificed by a lethal dose of pentobarbital sodium (90 mg·kg^-1^; Sigma-Aldrich, Saint Louis, MO, USA). Tissues (pancreas and colon) were excised, snap-frozen in liquid nitrogen and stored at -80 °C, or fixed in 4% paraformaldehyde for later analysis. For ELISA assays, tissues (pancreas and colon) were homogenized with phosphate-buffered saline and centrifuged (3000 g, 15 min); the supernatant was then collected and stored at -80 °C.

### Preparation of single-cell suspensions

When the mice were euthanized (at 12 weeks of age in NOD mice or at 27 days after STZ injection in STZ-induced diabetic mice), mesenteric lymph nodes (MLNs), pancreatic lymph nodes (PLNs), and pancreas were harvested and placed in cold PBS immediately. For macrophage (MΦ)/dendritic cells (DCs) detection, the fresh pancreas was cut into small pieces, digested with 0.75 mg·mL^-1^ collagenase P (Roche Basel, Switzerland) at 37 °C for 15 min, homogenized with gentle MACS™ Dissociators (MiltenyiBiotec, BergischGladbach, Germany) and filtered with 70 μm filter screen 15, 25. For T cells detection, the fresh pancreas was filtered with 70 μm polypropylene mesh along with grinding using a 2.5 mL syringe plunger and PBS (containing 10% fetal bovine serum) scouring 15, 25. PLNs and MLNs were filtered with 70 μm polypropylene mesh along with grinding using a 2.5 mL syringe plunger and PBS scouring 15, 25.

### Flow cytometry

At 12 weeks of age in NOD mice, or 27 days after STZ injection in STZ-induced diabetic mice, single-cell suspensions of the pancreas, PLNs, and MLNs were prepared 15, 25, stained for 30 min at 4 °C after FcγRII/III blocking with the anti-CD16/CD32 monoclonal antibody. The antibody information is listed in Table S1-2. For Foxp3^+^ regulatory T cells (Tregs) staining, cells were first surface stained, then fixed and stained for intracellular (nuclear) Foxp3 according to the manufacturer's protocol. For the detection of intracellular (cytoplasmic) cytokine interferon-gamma (IFN-γ) expression, cell suspensions were incubated at 37 °C for 5 h with a cell stimulation cocktail plus protein transport inhibitors of phorbol 12-myristate 13-acetate, ionomycin, brefeldin A and monensin (eBioscience, CA, USA), then cells were stained and fixed according to the manufacturer's protocol. Stained cells were analyzed on an Invitrogen™ Attune™ NxT Flow Cytometer (Thermo Fisher Scientific, Waltham, MA, USA).

### Histological evaluation

After the mice were sacrificed (at 12 weeks of age in NOD mice, or 27 days after STZ injection in STZ-induced diabetic mice), the fresh pancreas samples were fixed in NEG-50 (Thermo Scientific, MA, USA) and immediately stored at -80 °C until used for frozen sections, or fixed in 4% paraformaldehyde (Sigma, HT50-1-2, St. Louis, MO, USA) overnight, washed with ddH_2_O, rehydrated with gradient ethanol solutions and embedded in paraffin. 8 μm sections were stained with hematoxylin-eosin staining (H&E) following the standard procedure 7, 25. Fresh colon samples were fixed in 4% paraformaldehyde overnight, washed with ddH_2_O, rehydrated with gradient ethanol solutions, and embedded in paraffin. 5 μm sections were stained with H&E.

### Histomorphology of pancreas

Insulitis and insulitis score at 12 weeks of age in NOD mice. 1 = white, no infiltration; 2 = light gray, few mononuclear cells infiltrated; 3 = gray, peri-insulitis; 4 = dark gray, < 50% islet infiltration; 5 = black, >50% islet infiltration.

H&E staining was performed to observe pancreatic morphology in STZ-induced diabetic mice. 1 = white, no infiltration; 2 = light gray, mild inconsistent pancreatic islet cell size, islet atrophic, hyperplasia around the pancreatic duct and pancreatic duct expansion; 3 = gray, severe inconsistent pancreatic islet cell size, islet atrophic, hyperplasia around the pancreatic duct and pancreatic duct expansion.

### Histomorphology of colon

H&E staining was performed to observe colonic morphology. Score 0: normal colon mucosa with intact epithelium; Score 1: scattered inflammatory cell infiltrates in the mucosa; Score 2: diffuse mucosal infiltrate without submucosal spreading and intact epithelial layer; Score 3: moderate infiltration of inflammatory cells into mucosa and submucosa with epithelial hyperplasia and goblet cell loss; Score 4: marked inflammatory cell infiltrates in mucosa and submucosa accompanied by crypt abscesses and loss of goblet cells and crypts; Score 5: marked inflammatory cell infiltrates within the mucosa spreading to the submucosa going along with crypt loss and hemorrhage 26.

### Stool sampling, DNA extraction, and sequencing

3 weeks after STZ injection, feces samples were collected and stored at -80 °C immediately until used for the extraction of fecal microbial genomic DNA with Fast DNA Spin Kit for Soil (MP Biomedicals, cat. # 6560-200, CA, USA) following the manufacturer's instructions 25.

In detail, 50 mg of frozen stools were added to Lysing Matrix A tube, then 1 mL CLS-TC was added to Sample Tube. The mixture was homogenized in the FastPrep Instrument for 40 s at a speed setting of 6.0, then centrifuged at 14000 g for 5-10 min to pellet debris. Next, the supernatant was transferred to a 2 mL microcentrifuge tube, an equal volume of Binding Matrix was added, and the samples were mixed and incubated with gentle agitation for 5 min at room temperature on a rotator. The suspension was then transferred to a SPIN™, filtered, and centrifuged (14000 g, 1 min) twice. Subsequently, the pellet was resuspended gently with 500 µl prepared SEWS-M, and centrifuged (14000 g, 1 min). The contents of the Catch Tube were discarded, and the Catch Tube was replaced, centrifuged (14000 g, 1 min) without any addition of liquid. New, clean Catch Tubes were replaced, the Binding Matrix above the SPIN filter was resuspended with 100 µL DES to elute DNA, centrifuged at 14000 g for 1 min to bring eluted DNA into the clean catch tube after incubating the tubes at 55 °C for 5 min. The DNA is now ready for downstream applications and stored at -80 °C until use.

The V3, V4 region of 16S rRNA was PCR-amplified using specific primers. Reaction conditions were: 95 °C for 5 min; 95 °C for 30 s, 64 °C for 30 s, 72 °C for 30 s, repeated for 40 cycles, with a final incubation at 72 °C for 10 min. The PCR products were excised from a 1.5% agarose gel, purified by Gene Clean Turbo (MP Biomedicals, cat. # 111102400, CA, USA), and quantified by Quant-iTPicoGreen dsDNA Assay Kit (Life Technologies, cat. # P7589, CA, USA) following the manufacturer's instructions. Libraries were prepared using TruSeq DNA LT Sample Preparation Kit (Illumina, cat. # FC-121-2001, CA, USA) and sequenced for 500+7 cycles on Illumina MiSeq using the MiSeqReagent Kit (500 cycles-PE, cat. # MS-102-2003, CA, USA). The sequences reported in this paper have been deposited in the BioProject of NCBI under accession NO. PRJNA555462.

### Real-time PCR analysis

After the mice were euthanized (at 12 weeks of age in NOD mice, or 27 days after STZ injection in STZ-induced diabetic mice), total RNA was extracted from frozen tissues with TRIZOL reagent (Invitrogen, CA, USA), according to the manufacturer's instructions. Fast-Start SYBR Green PCR reagents (Roche, NSW, USA) were used to determine the mRNA level of CRAMP.

DNA extraction was performed as described above. The final step for conversion of butyryl-CoA to butyrate is either catalyzed by butyrate kinase or acetate CoA-transferase. Typically, these two genes are used as biomarkers for the identification/detection of butyrate-producing communities 27. Targeting the whole pathway for functional predictions is a robust way to circumvent difficulties associated with the analysis based on specific genes only 25, 28. Primer sequences are given in Table S3.

### Short-chain fatty acids (SCFAs) analysis

In stool samples, acetate, propionate, and butyrate were analyzed by gas chromatography coupled mass spectrometry (GC-MS) as previously described 15, 25. 3 weeks after STZ injection, stool samples were collected and immediately stored at -80 °C. Stool samples (50 mg) were first homogenized in 500 μL of saturated NaCl solution, then acidified with 40 μL of 10% sulfuric acid. After that, 1 mL diethyl ether was added to the samples to extract SCFAs, then samples were centrifuged at 14,000 g, 4 °C, 15 min, and the supernatant was used for GC-MS. 1 µL supernatants were injected into Rtx-WAX capillary column (30 m×0.25 mm×0.25 μm, Bellefonte, PA, USA) installed on the GC and coupled to the MS detector of GCMS-QP2010 (Shimadzu, Japan). The initial oven temperature was 100 °C, then increased to 140 °C at a rate of 7.5 °C·min^-1^. The temperature was further increased to 200 °C at a rate of 60 °C·min^-1^, remained for 3 min. The carrier gas was helium at a flow rate of 0.89 mL·min^-1^, and the column head pressure was 62.7 kPa. The injector was set at 240 °C. The injection mode was split, and the ratio was 10:1. For the mass spectrometer, the ion source temperature was 220 °C, the interface temperature was 250 °C, and the scan range was from m/z 2 to 100. Real-time analysis software GCMS Post run (GCMS solution Version 2.72) was used to compare the relative concentrations of the SCFAs.

### Enzyme-linked immunosorbent assays (ELISA)

After the mice were euthanized (at 12 weeks of age in NOD mice, or 27 days after STZ injection in STZ-induced diabetic mice), the tissues were harvested and homogenized with 20 mM phosphate buffer, the homogenate was centrifuged at 4 °C for 10 min at 12,000 g, and the supernatant was used for ELISA analysis. According to the manufacturer's instructions, the colonic and pancreatic CRAMP levels were measured by ELISA kit (CUSABIO BIOTECH CO., LTD, Hubei, China). The serum and pancreatic insulin levels were measured by ELISA kit (SenBeiJia Biological Technology Co., Ltd., Jiangsu, China).

### Western blot analysis

After the mice were euthanized (at 12 weeks of age in NOD mice, or 30 days after STZ injection in STZ-induced diabetic mice), the colon and pancreas samples were lysed with RIPA buffer containing protease inhibitors (Beyotime, Shanghai, China), ground with high-throughput tissue burnisher (SCIENTZ-48, Zhejiang, China). The homogenates were centrifuged at 4 °C for 15 min at 8000 g. The supernatant was used for Western blot. Protein concentration was quantified by BCA protein assay kit (Beyotime, Shanghai, China), equal amounts of total proteins were loaded on a polyacrylamide SDS-PAGE gel. Proteins were transferred to a PVDF membrane, blocked with blocking buffer for 1 h at room temperature, incubated overnight at 4 °C with appropriate antibodies. The antibodies' information is given in Table S4. The PVDF membranes were incubated with fluorescently labeled horseradish peroxidase (HRP)-conjugated secondary antibodies (1:5000) for 2 h at room temperature. Immunoreactivity was analyzed using Western Lightening Plus-ECL (Pierce, Rockford, IL, USA). β-actin was adopted as an internal standard to control for unwanted sources of variation, and relative protein expression values were expressed as “fold mean of the controls” by comparing to the corresponding control value, and the control value was normalized to 1.0. Protein expression levels were quantified with Image J.

### Data and statistical analysis

Studies were designed using randomization and blinded analysis. We selected all group sizes based on previously published data for similar experiments; we did not conduct sample size calculations for these experiments. All data were presented as mean ± standard error of mean (SEM) (n = 5-12) and analyzed by GraphPad Prism 7 software (San Diego, CA, USA). One-way analysis of variance (ANOVA) followed by Tukey's multiple-comparison test was performed to determine the significance among three or more groups. The *t*-test (two-tailed) was used for two independent groups. In multigroup studies with parametric variables, post hoc tests were conducted only if F in ANOVA achieved statistical significance (*p <* 0.05) and there was no significant variance in homogeneity. Optimization of 1% was performed using the unweighted pair group method with arithmetic averages clustering algorithm and by principal component analysis (PCoA) using Past v2.16. The sample size declared in the different experimental groups was the number of independent mice in each group, and statistical analysis was done using these independent values. For Western blot and real-time PCR analysis, the relative protein or mRNA expression values were expressed as “fold difference” by comparing to the corresponding control value, and the control value was normalized to 1.0. Potential outliers were tested using Grubbs' test. *p* < 0.05 was statistically significant.

## Results

### Colonic CRAMP production is defective in *C. rodentium*-accelerated T1D

**Figure 1:**
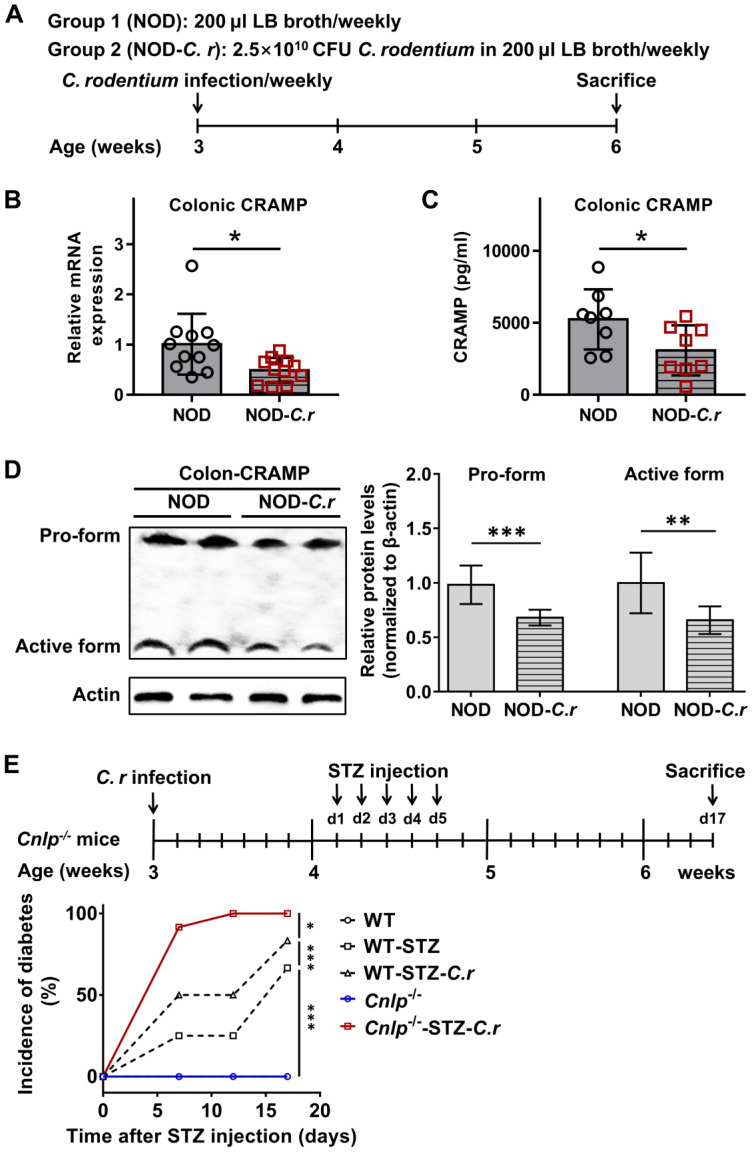
** Colonic CRAMP production is defective in *C. rodentium*-accelerated T1D. (A)** Animal protocol. Three-week-old female NOD mice were challenged with *C. rodentium* strain (2.5×10^10^ CFU in 200 µL LB broth) or 200 µL LB broth by gavage. Colonic CRAMP expression was determined by real-time PCR (**B**), ELISA (**C**), and Western blot (**D**), n = 8-11. **(E)** Animal protocol and incidence of diabetes. STZ-induced *Cnlp^-/-^* and WT diabetic mice were considered diabetic when glycemia was > 200 mg·dL^-1^ (11.1 mmol·L^-1^) after two consecutive determinations, n = 12. Data are mean ± SEM. * *p* < 0.05, ** *p* < 0.01, *** *p* < 0.001.

Murine CRAMP mRNA was constitutively expressed in the intestinal tract, where it is largely restricted to the colon 11. Interestingly, CRAMP expression was decreased after *C. rodentium* infection 11. We first examined how *C. rodentium* infection regulates colonic CRAMP production in diabetes-prone NOD mice (**Figure 1A-D**). Compared with NOD controls, *C. rodentium*-infected NOD mice exhibited significantly lower colonic CRAMP production at both mRNA and protein levels by real-time PCR and ELISA, respectively (**Figure 1B-C**). By further Western blot analysis, we found that both the pro-form (18KDa) and the active form (5KDa) of CRAMP were significantly reduced in *C. rodentium*-infected NOD mice as compared to NOD controls (**Figure 1D**). Similar results were also obtained in STZ-induced diabetic mice (**Figure S2**).

Interestingly, *C. rodentium* infection significantly accelerated the hyperglycemia in STZ-induced diabetes (**Figure S3**). More importantly, *Cnlp*^-/-^ diabetic mice were more susceptible to *C. rodentium*-accelerated T1D compared to the WT mice (**Figure 1E**). These data indicate that CRAMP plays an important role in the pathogenesis of *C. rodentium*-accelerated T1D.

### CRAMP protects against* C. rodentium*-accelerated T1D

**Figure 2:**
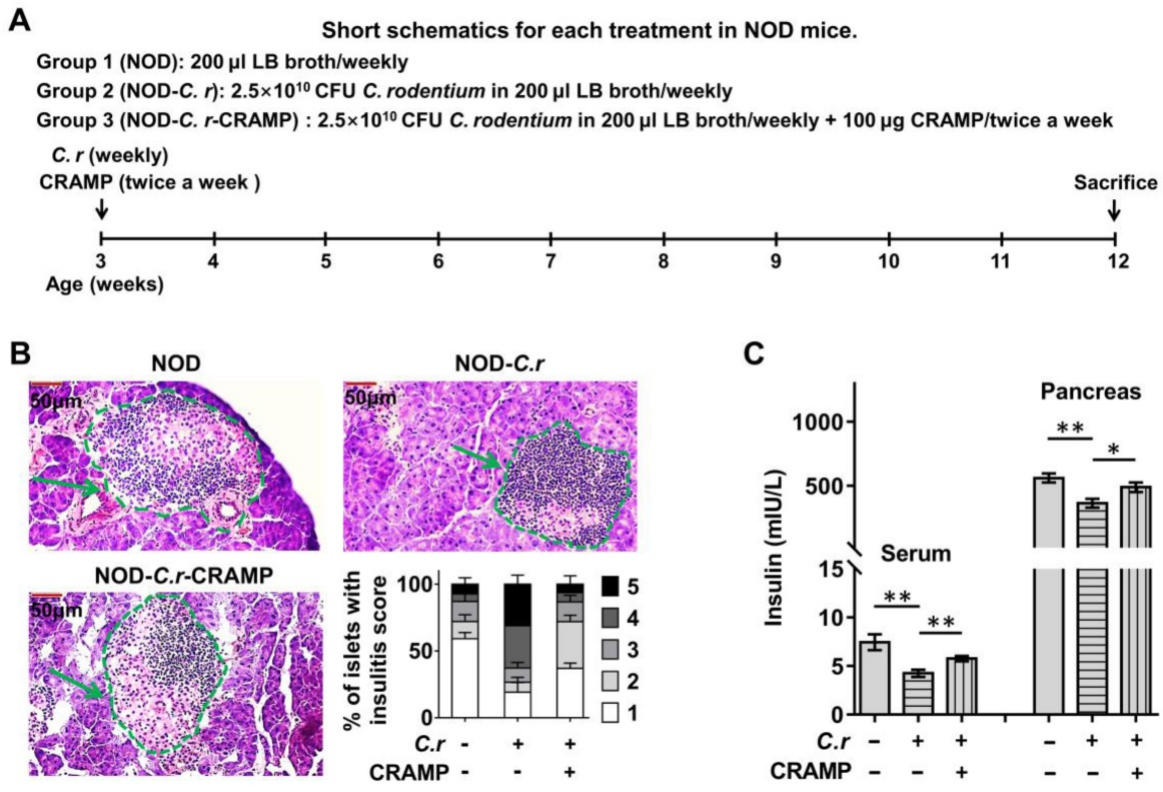
** CRAMP protects against *C. rodentium*-accelerated T1D in diabetes-prone NOD mice. (A)** Animal protocol. To evaluate the role and gut-pancreas pathophysiological mechanism of CRAMP in enteric pathogen-accelerated T1D, NOD mice were randomly assigned to three groups to receive CRAMP (2×100 µg) or equal volumes of saline by intraperitoneal injection twice a week after *C. rodentium* infection (2.5×10^10^ CFU in 200 µL LB broth) from 3 to 12 weeks of age. **(B)** Insulitis and insulitis score at 12 weeks of age in NOD mice. Scale bar: 50 µm. **(C)** Serum and pancreatic insulin levels were detected at 12 weeks of age in NOD mice. Data are mean ± SEM, n = 8. * *p* < 0.05, ** *p* < 0.01.

In diabetes-prone NOD mice, gut barrier disruption caused by enteric pathogen infection (including *C. rodentium*) accelerates the development of insulitis and T1D 4-6. To determine the consequences of gut barrier disruption in the T1D setting, following the previous study, we used the model enteric pathogen *C. rodentium* to simulate human enteropathogenic infection with gut barrier disruption in NOD mice 4, 11. The effect of CRAMP on enteric infection-complicated T1D was evaluated. In *C. rodentium*-infected NOD mice (**Figure 2A**), exogenous CRAMP treatment significantly inhibited the development of* C. rodentium*-exacerbated insulitis (**Figure 2B** and **Figure S4**), and reversed the *C. rodentium*-exacerbated reduction in insulin levels in both serum and pancreas (**Figure 2C**).

**Figure 3:**
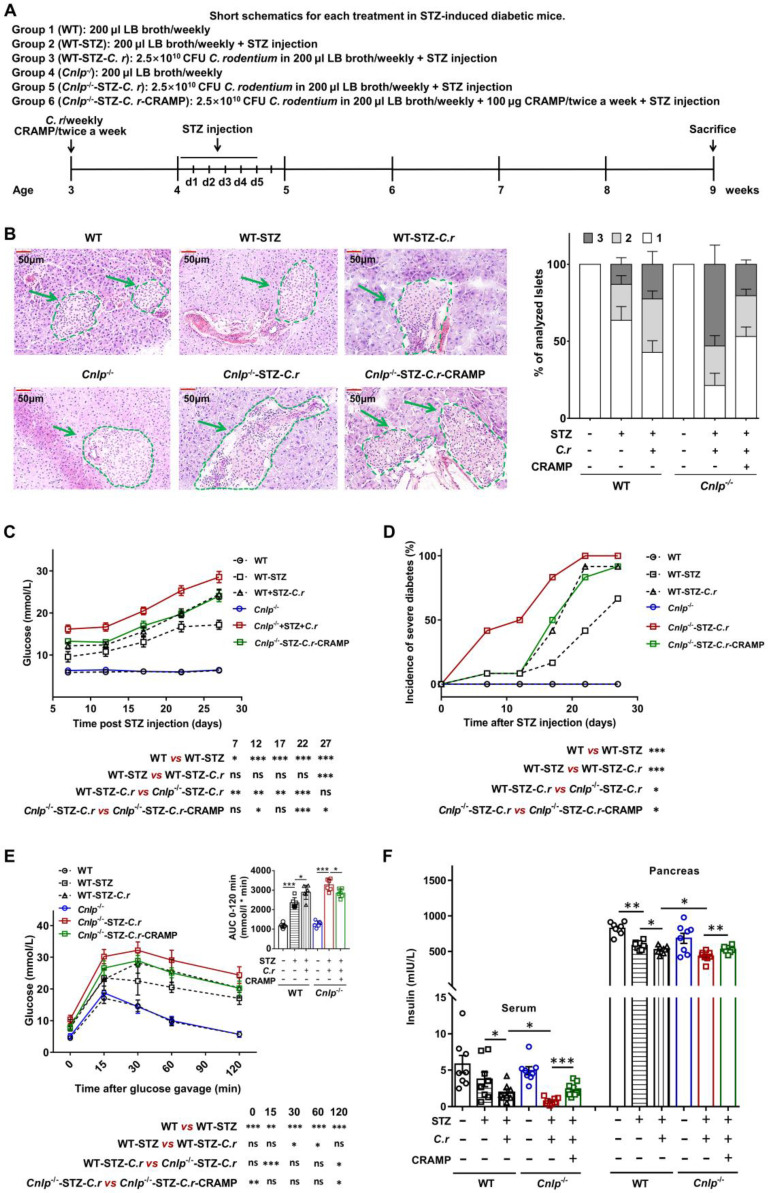
** CRAMP protects against *C. rodentium*-accelerated STZ-induced diabetes. (A)** Animal protocol. Three-week-old WT and *Cnlp^-/-^* mice were infected with the *C. rodentium* weekly along with the corresponding intervention as previously described from 3 to 9 weeks of age (n = 12). Infected mice were then injected with a low-dose of STZ by intraperitoneal injection for 5 consecutive days to induce T1D. All the mice received two injections of CRAMP (100 µg, twice a week) or saline by intraperitoneal injection twice a week. Mice were monitored for the glucose from the tail vein after 6 h fasting. **(B)** Insulitis and insulitis score in STZ-induced diabetic mice, n = 6. Scale bar: 50 µm. **(C)** Blood glucose was tested after 6 h fasting in STZ-induced diabetic mice, n = 12. **(D)** STZ-induced *Cnlp-/-* and WT diabetic mice were considered to have severe diabetes when glycemia was > 300 mg.dL^-1^ (16.6 mmol.L^-1^) after two consecutive determinations. **(E)** Glucose tolerance tests were performed after 18 h fasting at 3 weeks after STZ injection, n = 6. **(F)** Serum and pancreatic insulin levels were detected in STZ-induced diabetic mouse model at 3 weeks after STZ injection, n = 8. Data are mean ± SEM. * *p* < 0.05, ** *p* < 0.01, *** *p* < 0.001.

Subsequently, using STZ-induced diabetic CRAMP-deficient (*Cnlp*^-/-^) mice, the protective role of CRAMP in *C. rodentium*-accelerated diabetes was further confirmed (**Figure 3A**). Similarly, *C. rodentium* infection triggered more severe islet injury (evidenced by inconsistent islet cell size, islet atrophic, hyperplasia around the pancreatic duct and pancreatic duct expansion) in *Cnlp*^-/-^ diabetic mice than WT diabetic mice, which was significantly improved by exogenous CRAMP treatment (**Figure 3B** and **Figure S5**). Furthermore, *C. rodentium* infection resulted in higher fasting glucose levels (**Figure 3C**), severe diabetes incidence (**Figure 3D**), and further impaired glucose tolerance (**Figure 3E**) in* Cnlp*^-/-^ diabetic mice than WT diabetic mice. Exogenous CRAMP treatment significantly reversed these *C. rodentium* infection-exacerbated glucose intolerance and hyperglycemia in *Cnlp*^-/-^ diabetic mice (**Figure 3C-E**). To assess whether the hyperglycemia caused by CRAMP deficiency is a consequence of insufficient insulin production, both serum and pancreatic insulin levels were measured. As shown in **Figure 3F**, exogenous CRAMP treatment significantly inhibited *C. rodentium*-exacerbated reduction in serum and pancreatic insulin levels in STZ-induced *Cnlp*^-/-^ diabetic mice.

### CRAMP attenuates colonic barrier disruption and gut dysbiosis in *C. rodentium*-accelerated T1D

**Figure 4:**
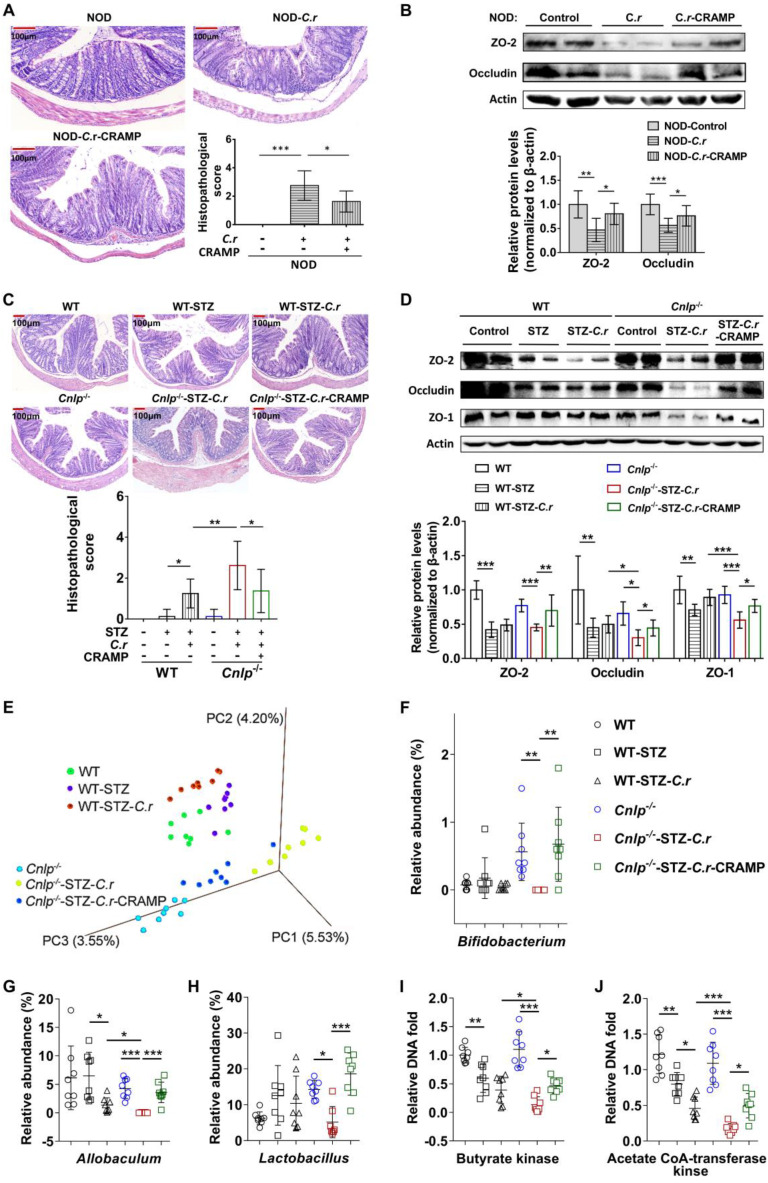
** CRAMP attenuates colonic barrier disruption and gut dysbiosis in *C. rodentium*-accelerated T1D. (A)** H&E staining of the colon and histopathological score in NOD mice. Scale bar: 100 µm. **(B)** Colonic tight junction protein (ZO-2 and occludin) determined by Western blot in NOD mice, and grey value analysis of western blot by Image J. **(C)** H&E staining of the colon and histopathological score in STZ-induced diabetic mice. Scale bar: 100 µm. **(D)** Colonic tight junction protein (ZO-2, occludin, and ZO-1) determined by Western blot in STZ-induced diabetic mice, and grey value analysis of Western blot by Image J. **(E)** PCoA plot of weighted UniFrac distances in STZ-induced WT and *Cnlp^-/-^* diabetic mice, each dot representing a colonic community; the percentage of variation explained by each principal coordinate is shown in parentheses. **(F)** The relative abundance of *Bifidobacterium* in STZ-induced WT and *Cnlp^-/-^* diabetic mice. **(G)** The relative abundance of *Allobaculum* in STZ-induced WT and *Cnlp^-/-^* diabetic mice. **(H)** The relative abundance of *Lactobacillus* in STZ-induced WT and *Cnlp^-/-^* diabetic mice. **(I)** Butyrate-producing genes: the abundance of butyrate kinase DNA in feces of STZ-induced *Cnlp^-/-^* and WT diabetic mice. **(J)** Butyrate-producing genes: the abundance of acetate CoA-transferase DNA in feces of STZ-induced WT and *Cnlp^-/-^* diabetic mice. Data are mean ± SEM, n = 8. * *p* < 0.05, ** *p* < 0.01, *** *p* < 0.001.

Enteric pathogen infection resulted in gut barrier disruption 4. CRAMP is essential for colon homeostasis 29. Consistent with earlier reports 4, 29, in diabetes-prone NOD mice, *C. rodentium* infection also resulted in severe colonic barrier disruption (characterized by goblet cell depletion, inflammatory cell infiltration, crypt cell hyperplasia, and thicker mucosa), which was significantly attenuated by CRAMP treatment (**Figure 4A**). Furthermore, in NOD mice, CRAMP treatment significantly enhanced the reduced expression of colonic tight junction proteins (TJPs, including ZO-2 and occludin) caused by *C. rodentium* infection, suggesting the improved colonic barrier function (**Figure 4B**). The STZ-induced *Cnlp*^-/-^ diabetic mouse model was used to further ascertain the causality between CRAMP and the improved colonic barrier disruption in *C. rodentium*-accelerated diabetes. Compared with *C. rodentium*-infected WT diabetic mice, the* C. rodentium*-infected* Cnlp*^-/-^ diabetic mice exhibited further impaired colon morphology and lower colonic TJPs (ZO-2, occludin, and ZO-1) protein levels, which were significantly reversed by CRAMP treatment (**Figure 4C*-*D**).

Gut barrier disruption caused by enteric pathogen infection leads to gut dysbiosis 4, 16. CRAMP is essential for colon homeostasis by maintaining intestinal microbial homeostasis 29, which is involved in the disease pathogenesis of T1D 30-32. AMPs shape the microbiome 16. Lack of pancreatic antimicrobials also disrupts the gut microbiome homeostasis, revealing a critical role for antimicrobial secretion in pancreatic-intestinal crosstalk 16. Therefore, we examined whether and how CRAMP modulated gut dysbiosis in *C. rodentium*-accelerated diabetes by 16S rRNA-based bacterial analysis. PCoA showed that the overall composition of gut microbiota was significantly modified by *C. rodentium* infection and CRAMP treatment (**Figure 4E**). In *Cnlp*^-/-^ diabetic mice, CRAMP treatment shifted *C. rodentium-*worsened adverse variations toward the direction of *Cnlp*^-/-^ control mice, indicating that CRAMP reverses the gut dysbiosis caused by* C. rodentium* infection to some extent (**Figure 4E** and **Figure S6-7**). Specifically, CRAMP effectively reversed the decrease in genus* Bifidobacterium* and *Allobaculum* (a potential butyrate producer) in* C. rodentium*-accelerated diabetes (**Figure 4F-G**) 33. Notably, CRAMP enriched *Lactobacillus* (**Figure 4H**), which is beneficial to T1D 34-36. By determining the gene biomarkers of butyrate-producing communities (butyrate kinase and acetate CoA-transferase), we observed that CRAMP treatment reversed the reduction of butyrate-producing communities in* C. rodentium*-accelerated diabetes (**Figure 4I*-*J**). Further GC-MS analysis showed that CRAMP significantly inhibited the reduction of fecal butyrate in* C. rodentium*-accelerated diabetes (**Figure S8**). In *Cnlp^-/-^* diabetic mice, CRAMP deficiency further exacerbated the gut dysbiosis caused by *C. rodentium* infection, while exogenous CRAMP treatment significantly reversed these CRAMP deficiency-exacerbated adverse variations (**Figure S6*-*7**). In *C. rodentium*-accelerated diabetes, CRAMP-treated mice harbored more microbial clusters previously described as beneficial to T1D and fewer microbial clusters previously described as pathogenic to T1D than the untreated mice. Specifically, CRAMP promoted the establishment of a protective microbiome enriched in *Bifidobacterium*, *Lactobacillus,* and butyrate-producing communities.

### CRAMP attenuates gut immune dysregulation in *C. rodentium*-accelerated T1D

Gut barrier disruption caused by enteric pathogen infection results in intestinal and pancreatic immune dysregulation 4. Apart from PLNs, diabetogenic T cells are also primed in gut-associated lymphoid tissues (GALTs) in the T1D setting 37. Thus, whether and how CRAMP modulates gut immune dysregulation was also examined in the MLNs.

**Figure 5:**
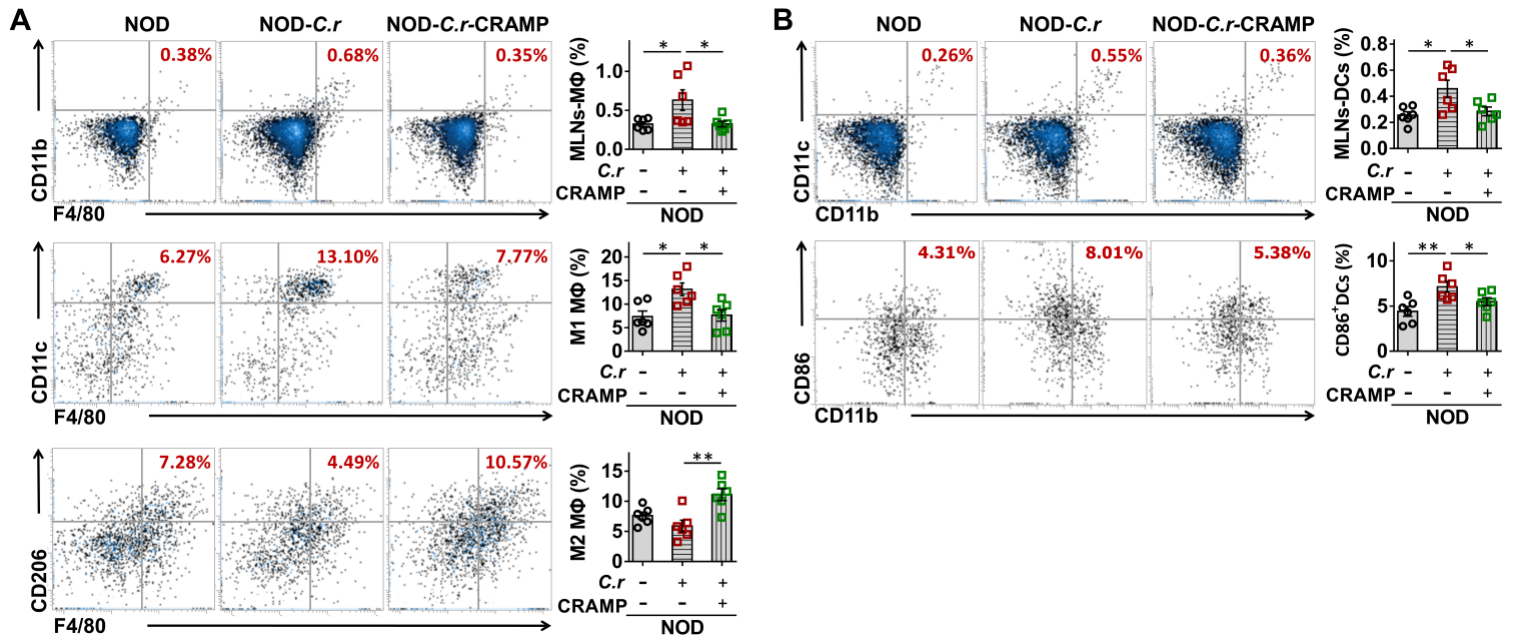
** CRAMP attenuates gut immune dysregulation in *C. rodentium*-accelerated T1D. (A)** The percent of F4/80^+^CD11b^+^ cells in CD45^+^ cells (total Mφ) in MLNs (top), F4/80^+^CD11b^+^CD11c^+^ Mφ (M1 Mφ) and F4/80^+^CD11b^+^CD206^+^Mφ (M2 Mφ) in the total Mφ in MLNs (center and bottom) from the NOD mice. **(B)** The percent of CD11b^+^CD11c^+^ cells in CD45^+^ cells (DCs) in MLNs (top), and CD86 expression in DCs in MLNs (the percent of CD86^+^CD45^+^CD11b^+^CD11c^+^ cells in DCs from MLNs) (bottom) from the NOD mice. Data are mean ± SEM, n = 6. * *p* < 0.05, ** *p* < 0.01.

**Figure 6:**
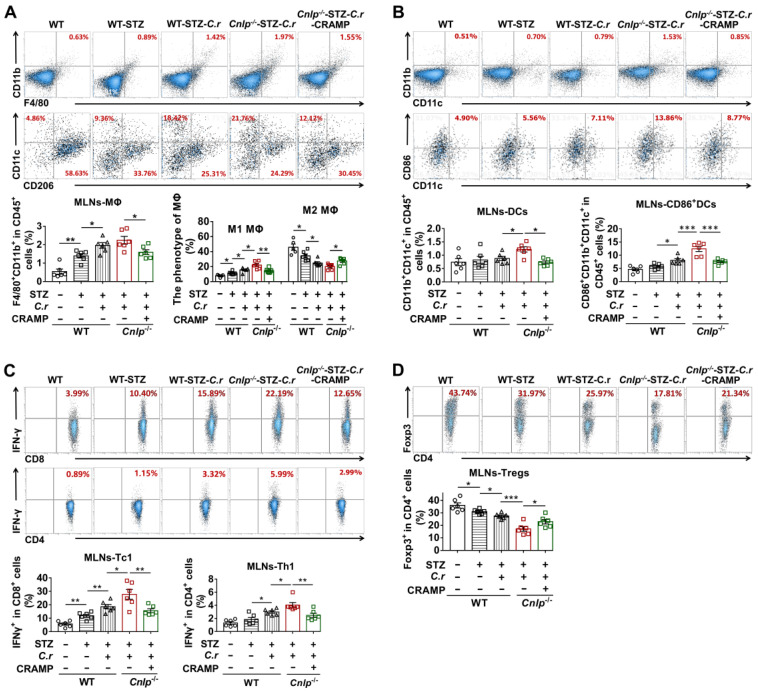
**CRAMP attenuates gut immune dysregulation in *C. rodentium*-accelerated STZ-induced diabetes.** (**A**) The percent of F4/80^+^CD11b^+^ cells in CD45^+^ cells (total MΦ) in MLNs (top) from STZ-induced diabetic mice, and the percent of CD11c^+^CD206^-^ MΦ (M1 MΦ) and CD206^+^CD11c^-^ MΦ (M2 MΦ) in the total MΦ in MLNs (bottom) from STZ-induced diabetic mice. (**B**) The percent of CD11b^+^CD11c^+^ cells in CD45^+^ cells (DCs) in MLNs (top), and CD86 expression in DCs in MLNs (bottom) from STZ-induced diabetic mice. (**C**) The percent of IFN-γ^+^ cells in CD8^+^ cells (Tc1) (top), the percent of IFN-γ^+^ cells in CD4^+^ cells (Th1) (bottom) in MLNs from STZ-induced diabetic mice. (**D**) The percent of Foxp3^+^ cells in CD4^+^ cells (Tregs) in MLNs from STZ-induced diabetic mice. Data are mean ± SEM, n = 6. * *p* < 0.05, ** *p* < 0.01, *** *p* < 0.001.

In the MLNs from diabetes-prone NOD mice, we observed increased frequencies of total MΦ and conventional DCs in response to *C. rodentium* infection, which was significantly attenuated by CRAMP treatment (**Figure 5A-B**). Furthermore, CRAMP treatment modulated the phenotype of macrophages and DCs in the MLNs, supporting that the pro-inflammatory effects of *C. rodentium* infection in diabetes-prone NOD mice were significantly reversed by subsequent CRAMP treatment (**Figure 5A-B**). Specifically, in NOD mice, *C. rodentium* infection increased the frequency of inflammatory macrophage and CD86^+^ cDCs in the MLNs, while CRAMP treatment significantly inhibited these adverse events (**Figure 5A-B**). Consistent with previous results in NOD mice, CRAMP treatment significantly reduced the frequencies of total MΦ and conventional DCs in MLNs from CRAMP-deficient (*Cnlp*^-/-^) diabetic mice (**Figure 6A-B**). Furthermore, CRAMP treatment inhibited increases in the ratio of inflammatory macrophages (M1 MΦ) versus regulatory macrophages (M2 MΦ) (**Figure 6A**) and the frequency of CD86^+^ cDCs in* C. rodentium*-accelerated diabetes (**Figure 6B**). Moreover, *C. rodentium* infection contributed to the expansion of IFN-γ^+^ T cells (cytotoxic T cells type 1 [Tc1] and T helper type 1 cells [Th1]) (**Figure 6C**) and the reduction of protective Tregs in the MLNs in* C. rodentium*-accelerated diabetes (**Figure 6D**). These T cell dysregulations caused by* C. rodentium* infection were further aggravated in *Cnlp*^-/-^ diabetic mice and were reversed by exogenous CRAMP treatment (**Figure 6C-D**). Collectively, CRAMP inhibits gut immune dysregulation, especially IFN-γ^+^ T cell recruitment in* C. rodentium*-accelerated diabetes.

### CRAMP inhibits the migration of gut-primed IFN-γ^+^ T cells to the pancreas in *C. rodentium*-accelerated T1D

**Figure 7:**
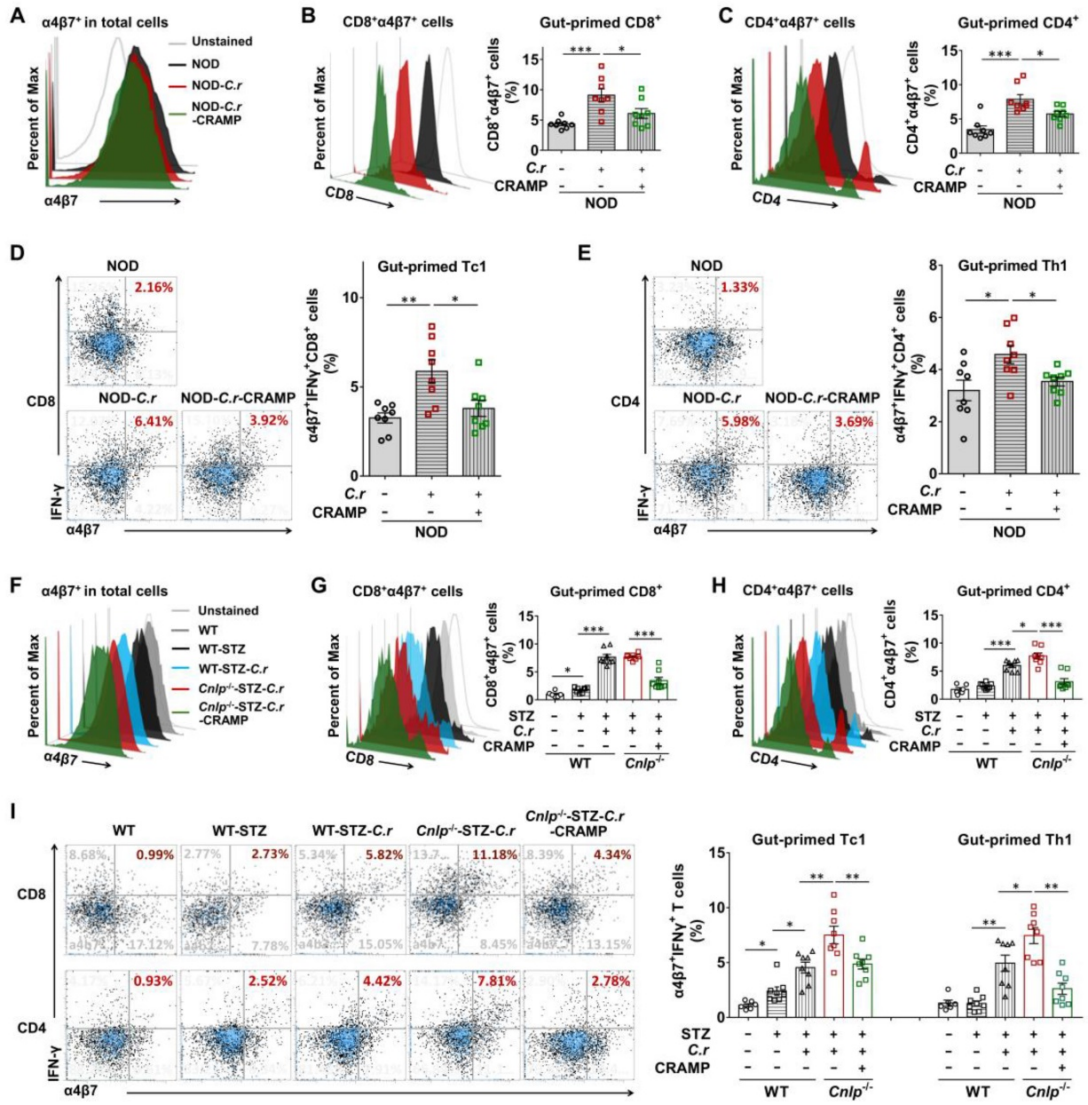
**CRAMP inhibits migration of gut-primed IFN-γ^+^ T cells to the pancreas in *C. rodentium*-accelerated T1D.** (**A**) The percent of α4β7^+^ cells in total pancreatic cells (gut-primed cells) from NOD mice. (**B**) The percent of α4β7^+^CD8^+^ cells in α4β7^+^ cells (gut-primed CD8^+^ T cells) in the pancreas from NOD mice. (**C**) The percent of α4β7^+^CD4^+^ cells inα4β7^+^ cells (gut-primed CD4^+^ T cells) from the pancreas of NOD mice. (**D**) The percent of α4β7^+^CD8^+^IFN-γ^+^ cells in CD8^+^cells (gut-primed Tc1) from the pancreas of NOD mice. (**E**) The percent of α4β7^+^CD4^+^IFN-γ^+^ cells in CD4^+^ cells (gut-primed Th1) from the pancreas of NOD mice. (**F**) The percent of α4β7^+^ cells in total pancreatic cells (gut-primed cells) from STZ-induced diabetic mice. (**G**) The percent of α4β7^+^CD8^+^ cells in α4β7^+^ cells (gut-primed CD8^+^ T cells) in the pancreas of STZ-induced diabetic mice. (**H**) The percent of α4β7^+^CD4^+^ cells in α4β7^+^ cells (gut-primed CD4^+^ T cells) from the pancreas of STZ-induced diabetic mice. (**I**) The percent of α4β7^+^CD8^+^IFN-γ^+^ cells in CD8^+^ cells (gut-primed Tc1) from the pancreas of STZ-induced diabetic mice; the percent of α4β7^+^CD4^+^IFN-γ^+^ cells in CD4^+^ cells (gut-primed Th1 cells) from the pancreas of STZ-induced diabetic mice. Data are mean ± SEM. n = 6-8. * *p* < 0.05. * *p* < 0.05, ** *p* < 0.01, *** *p* < 0.001.

Unlike tissue-resident cells such as MΦ and DCs, T cells are rarely found in healthy islets. Thus, the recruitment of diabetogenic effector T cells to the pancreas is a critical step for islet inflammation and beta-cell destruction in T1D 38. Loss of gut barrier continuity leads to the activation of diabetogenic T cells within the intestinal mucosa and to T1D, suggesting the presence of pancreatic-intestinal crosstalk 2. Therefore, we evaluated whether CRAMP affected intestinal T cell migration to the pancreas by detecting the frequency of pancreatic T cells expressing α4β7 integrin (a gut homing marker) 2, 39. We observed the presence of α4β7^+^ cells in the pancreas with or without *C. rodentium* infection in both NOD mice and STZ-induced diabetic mice (**Figure 7A and F**). The frequency of gut-primed cells (α4β7^+^ cells) in the pancreas responded to neither *C. rodentium* infection nor CRAMP treatment (**Figure 7A and F**). Furthermore, the frequencies of pancreatic α4β7^+^ cells in WT and NOD mice were similar (**Figure S9A**). Therefore, the trafficking pattern of intestinally primed T cells to the pancreas may be a general phenomenon, not particular to ongoing intestinal infection, pancreatic inflammation, or diabetes-prone NOD mice. Intriguingly, the NOD mice harbored higher frequencies of pancreatic α4β7^+^CD8^+^ and α4β7^+^CD4^+^ T cells than healthy WT control mice (**Figure S9B*-*C**).

More importantly, compared with healthy WT mice, the pancreatic α4β7^+^CD8^+^ and α4β7^+^CD4^+^ T cells from diabetes-prone NOD mice showed an increased IFN-γ-secreting phenotype (**Figure S9E*-*F**).

Notably, the *C. rodentium*-infected NOD mice showed higher frequencies of pancreatic α4β7^+^CD8^+^ cells and pancreatic α4β7^+^CD4^+^ cells than NOD controls, which were effectively suppressed by CRAMP treatment (**Figure 7B-C**). More interestingly, pancreatic α4β7^+^CD8^+^ and α4β7^+^CD4^+^ T cells from the *C. rodentium-*infected NOD mice showed an increased IFN-γ-secreting phenotype compared to the NOD controls, which were also significantly inhibited by exogenous CRAMP treatment (**Figure 7D-E**).

The roles of CRAMP in the migration of gut-primed T cells to the pancreas were further investigated in STZ-induced *Cnlp*^-/-^ diabetic mice. Consistent with previous results in NOD mice, in STZ-induced diabetic mice, the WT diabetic mice harbored higher frequencies of α4β7^+^CD8^+^ and α4β7^+^CD4^+^ T cells than the WT control mice. CRAMP deficiency further aggravated increases in the frequencies of pancreatic α4β7^+^CD8^+^ and α4β7^+^CD4^+^ cells in* C. rodentium*-accelerated diabetes, which were significantly inhibited by exogenous CRAMP treatment (**Figure 7G-H**). Most importantly, in STZ-induced diabetic mice infected with *C. rodentium*, CRAMP deficiency further enhanced the abundance of pancreatic α4β7^+^CD8^+^IFN-γ^+^ T and α4β7^+^CD4^+^IFN-γ^+^ T cells (**Figure 7I**), while CRAMP treatment effectively reversed the CRAMP deficiency-aggravated infiltration of α4β7^+^IFN-γ^+^ T cells in the pancreas (**Figure 7I**).

Apart from the pancreas, similar results were also obtained in PLNs (**Figure S10**), where the autoimmune that cascade culminates in diabetes initiates 40. Together, these data indicate that CRAMP inhibits migration of gut-primed IFN-γ^+^ T cells into the pancreas and its draining lymph nodes in* C. rodentium*-accelerated T1D.

### CRAMP attenuates pancreatic immune dysregulation in *C. rodentium*-accelerated diabetes

**Figure 8:**
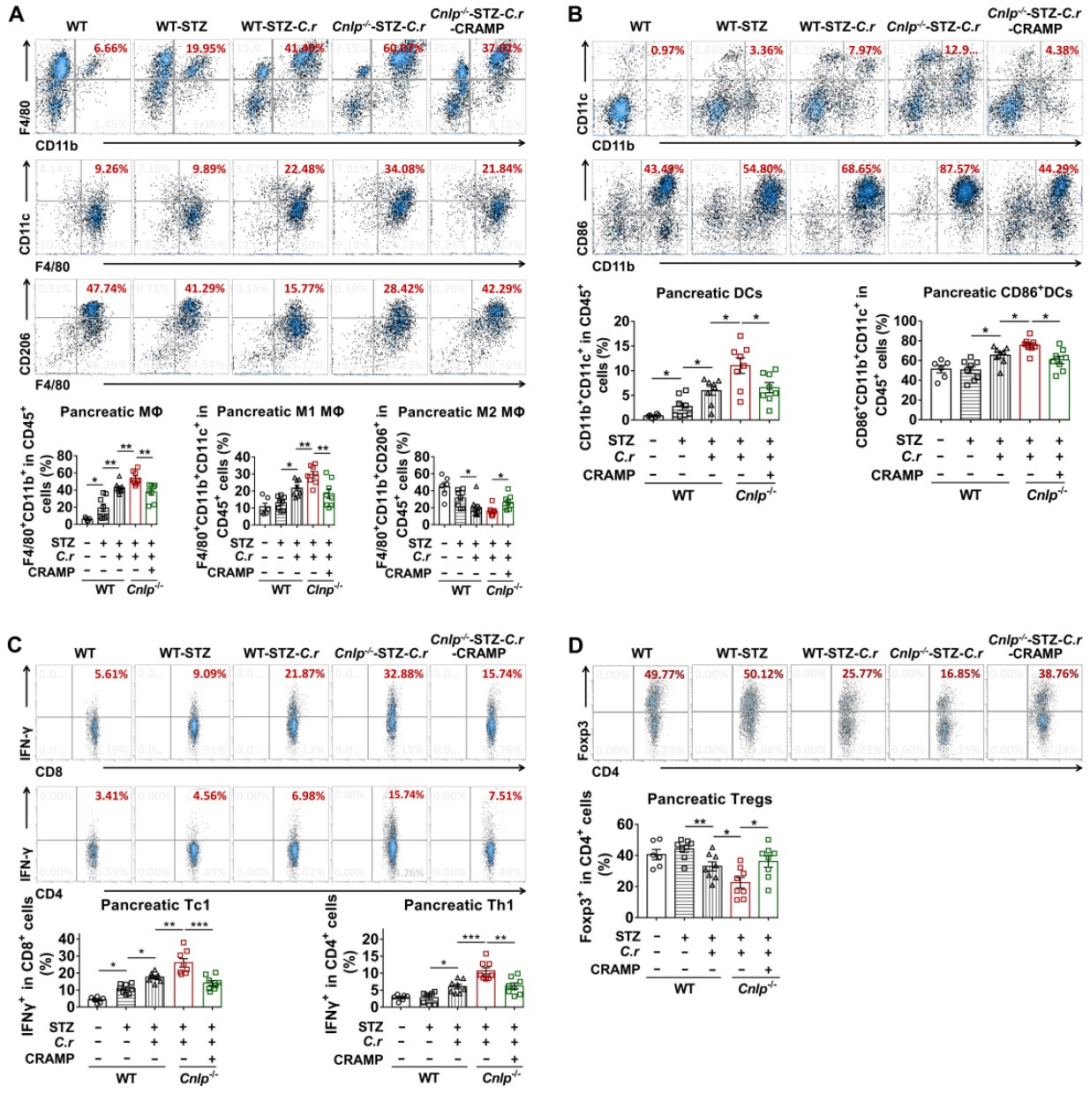
**CRAMP attenuates pancreatic immune dysregulation in *C. rodentium*-accelerated diabetes.** (**A**) The percent of MΦ (top) in the pancreas, the percent of CD11c^+^CD206^-^ MΦ (M1 MΦ) (center) and CD206^+^CD11c^-^ MΦ (M2 MΦ) (bottom) in total MΦ in the pancreas of STZ-induced diabetic mice. (**B**) The percent of CD45^+^CD11b^+^CD11c^+^ cells in CD45^+^ cells (total DCs) and CD86^+^ DCs in the pancreas from STZ-induced diabetic mice. (**C**) The percent of CD8^+^IFN-γ^+^ cells in CD8^+^ cells (Tc1) (top), CD4^+^IFN-γ^+^ cells in CD4^+^ cells (Th1 cells) (bottom) in the pancreas from STZ-induced diabetic mice. (**D**) The percent of CD4^+^Foxp3^+^ cells from in CD4^+^ cells (Tregs) in the pancreas from STZ-induced diabetic mice. Data are mean ± SEM, n = 6-8. * *p* < 0.05, ** *p* < 0.01, *** *p* < 0.001.

The gut immune environment influences pancreas-directed autoimmunity 40. In *C. rodentium*-accelerated diabetes, apart from the colon, we observed that intestinal *C. rodentium* infection also resulted in defective production of pancreatic CRAMP (**Figure S11**). We then examined whether CRAMP modulated pancreatic immune dysregulation using CRAMP defective *Cnlp*^-/-^ diabetic mice. In the pancreas,* C. rodentium* infection significantly enhanced the infiltration of total F4/80^+^CD11b^+^ MΦ (**Figure 8A**), M1/M2 MΦ ratio (**Figure 8A**), and the infiltration of pancreatic cDCs as well as CD86^+^ cDCs (**Figure 8B**). The effects of *C. rodentium* infection on antigen-presenting cells (APCs) (MΦ and DCs) were further exacerbated in *Cnlp^-/-^* diabetic mice and were reversed by exogenous CRAMP treatment (**Figure 8A-B**). In the pancreas from WT diabetic mice, *C. rodentium* infection significantly exacerbated the recruitment of diabetogenic IFN-γ^+^ T cells and the reduction of protective Tregs (**Figure 8C-D**). More importantly, *C. rodentium*-infected* Cnlp^-/-^* diabetic mice harbored a higher proportion of diabetogenic IFN-γ^+^ T cells and a lower proportion of protective Tregs than *C. rodentium*-infected WT diabetic mice (**Figure 8C-D**). These adverse effects of *C. rodentium* infection on T1D-associated T cells (IFN-γ^+^ T cells and Tregs) in *Cnlp*^-/-^ diabetic mice were effectively reversed by exogenous CRAMP supplement (**Figure 8C-D**).

## Discussion

**Figure 9:**
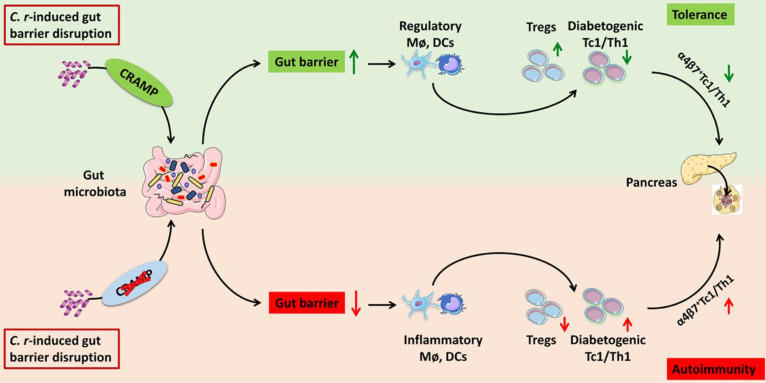
** Graphical abstract-schematic representation of the underlying mechanisms by which CRAMP protects against enteric pathogen-accelerated T1D.** During *C. rodentium*-accelerated T1D, CRAMP plays a beneficial role in pancreatic-gut crosstalk by gut barrier-protective, immune- and microbial-modulatory mechanisms, thus protecting against T1D.

T1D may originate in the intestine, and disruption of intestinal barrier integrity is involved in the pathogenesis of T1D 2, 4, 41. Here, we show that CRAMP plays a pivotal role in pancreatic-intestinal crosstalk during *C. rodentium*-accelerated T1D by barrier-protective, microbial- and immune-modulatory mechanisms. In the case of intestinal barrier disruption caused by enteric pathogen (*C. rodentium*) infection, genetic ablation of CRAMP further promotes the gut barrier disruption, intestinal IFN-γ^+^ T cell recruitment and their migration to the pancreas, ultimately exacerbating diabetes. Conversely, the use of CRAMP to restore a healthy intestinal barrier inhibits intestinal IFN-γ^+^ T cells recruitment and their migration to the pancreas, thus protecting against T1D (**Figure 9**). More interestingly, the efficacy of CRAMP by intracolonic administration on *C. rodentium*-accelerated insulitis was further confirmed its effectiveness in the gut (**Figure S12-14**).

It has been earlier demonstrated that* C. rodentium* could accelerate T1D development by impairing the gut barrier 4. CRAMP exerts anti-bacterial effects on *C. rodentium* 11, 42. Here in the present study, we focused on the additional immunomodulatory effects of CRAMP on *C. rodentium*-accelerated T1D, other than its direct anti-bacterial effects. T1D is primarily caused by the selective destruction of pancreatic insulin-producing beta-cells induced by diabetogenic IFN-γ^+^ T cells. T cells are rarely found in healthy islets. Thus, the recruitment of diabetogenic effector T cells, especially IFN-γ^+^ T cells, to the pancreas is a critical step to initiate T1D 38, 43. Developmentally controlled lymphogenesis establishes a preferential trafficking of intestinal cells to the pancreas and its draining lymph nodes, where T cells can be activated by antigens/microbiota drained from the gastrointestinal tract 40. Interestingly, apart from the pancreas and PLNs, the primary activation of diabetogenic T cells also occurs within GALTs, suggesting the presence of intestinal-pancreatic crosstalk 37, 44, 45. Furthermore, gut barrier disruption leads to the activation of diabetogenic effector T cells within the intestinal mucosa and to the development of T1D 2, 39. More interestingly, gut dysbiosis triggers intestinal immune responses and activates intestinal APCs, thus promoting the proliferation of intestinal antigen-specific IFN-γ^+^ T cells in GALTs 4, 41 and the migration of intestinal T cells to the pancreas 2, 39. CRAMP is an essential component of innate antimicrobial defense in the colon. Intestinal CRAMP reduces immune cell infiltration caused by colitis 12 and protects against *C. rodentium*-induced colonic epithelial barrier dysfunction 4, 11. Even more impressively, CRAMP maintains intestinal barrier integrity, microbial homeostasis, and dampens the development of pancreatic diseases 15, 16. Lack of antimicrobial peptides disrupts the gut microbiome homeostasis and accelerates T1D development 15, 16. However, whether and how CRAMP plays a role in pancreatic-gut crosstalk remains unknown. To test whether CRAMP-mediated gut immune defense contributes to reduced intestinally primed T cells in the pancreas, we evaluated gut-primed T cells in the pancreas by detecting the frequency of pancreatic T cells expressing α4β7 integrin (a gut homing marker) 2, 39. Previous studies have demonstrated the preferential trafficking of intestinal cells to the pancreas/PLNs 40. Our data presented the correlation among reduced CRAMP, gut barrier disruption, and the increased risk of T1D. Gut barrier disruption caused by *C. rodentium* infection may facilitate the priming of intestinal IFN-γ^+^ T cells and their migration to the pancreas, promote the otherwise sluggish response of pancreatic T cells to the islet autoantigens by increasing intestinal APCs (mainly MΦ and DCs) and exerting a negative immune-regulatory effect on these APCs. These effects were further exacerbated in CRAMP deficient *Cnlp*^-/-^ mice, while CRAMP treatment efficiently strengthened gut defense, inhibited these adverse processes, and dampened T1D development. Thus, restoring a healthy gut barrier by CRAMP may inhibit the proliferation of intestinal IFN-γ^+^ T cells and their migration to the pancreas.

Gut microbiota is a target for improving the outcomes of T1D 46. Enteric pathogen during gut dysbiosis may drive the establishment of lymphatic connections between the intestine and pancreas as well as the increased supply of self-antigens by affecting immunological or metabolic signals. Both colonic and pancreatic CRAMP are essential for innate antimicrobial defense, intestinal barrier integrity, and microbial homeostasis 12, 16. Lack of CRAMP disrupts the gut microbiome homeostasis, revealing a critical role for CRAMP in the regulation of intestinal microbiome 12, 16. Decreased *Bifidobacterium* was found in children with T1D 47. Furthermore, *Bifidobacterium* and *Lactobacillus* play crucial roles in strengthening the gut barrier, exerting anti-inflammatory effects and antimicrobial activities that participate in host gastrointestinal defense system through cytokine secretion or butyrate production 34, 46-48. As the preferred energy source for colon epithelial cells, butyrate is essential for maintaining intestinal barrier functions and has potent immunoregulatory effects on intestinal immune cells, especially T cells 49, 50. Lack of butyrate-producing bacteria or butyrate impairs the integrity of the gut barrier, function of the mucosa and intestinal T cell responses 49, 50, and has been considered a contributing factor of T1D 25, 51. Thus, in the present study, it could be speculated that the reduced *Bifidobacterium*,* Lactobacillus,* butyrate-producing bacteria and its metabolite butyrate caused by *C. rodentium* infection (**Figure 4** and **Figure S8**) may facilitate the progression of T1D by disrupting gut barrier integrity. The selective modulation of gut microbial phenotypes by CRAMP treatment, particularly the enrichment of *Bifidobacterium, Lactobacillus*, and butyrate producers, may enhance the gut barrier function and maintain intestinal immune tolerance (**Figure 4** and **Figure S8**). Intriguingly, oral administration of *Bifidobacterium, Lactobacillus,* or butyrate-producing bacteria in early diabetic NOD mice has been shown to prevent islet destruction and the onset of clinical signs of T1D 25, 35. More importantly, *Bifidobacterium*, *Lactobacillus,* and butyrate producers are widely used in clinical practice to treat patients with gut dysbiosis-associated diseases (such as colitis, constipation, diarrhea, and eczema), which are associated with the activation of diabetogenic T cells in GALTs and accelerated T1D 52. Furthermore, these three probiotics are also widely used in the food industry to produce the combined fermented dairy products, including cheese and yogurt, which have been known to be beneficial for glycaemic control and inversely associated with T1D 53, 54. Collectively, our study presents the possibility of targeting cathelicidin to modify the gut microbiome profile to prevent T1D in diabetes-prone individuals.

MyD88/NF-κB-NLRP3 pathway is critically involved in initiating immune responses to enteric pathogens that may contribute to T1D development 38, 55-58. Previous studies using MyD88^-/-^ NOD mice suggest the intimate three-way relationship among commensal bacteria, MyD88, and T1D predisposition 59, 60. Interestingly, exogenous CRAMP/IL-37 at physiological concentrations aborts MyD88 synthesis and its interaction with IRAK-4, which is involved in the activation of NF-κB/MAPKs 61, 62 and NLRP3 signaling 63. NLRP3 inflammasome, as the downstream signaling protein of MyD88, has been required for the secretion of IL-1β and IL-18 57, 58. More interestingly, inhibition of NLRP3 suppresses the activation of T cells and the differentiation of IFN-γ^+^ Th1 cells and impairs the migration of diabetogenic T cells to the pancreas 38. In* C. rodentium*-accelerated T1D, the activation of MyD88/NF-κB-NLRP3 pathway was further enhanced in CRAMP defective* Cnlp*^-/-^ diabetic mice, while CRAMP treatment significantly inhibited MyD88/IRAK-4/TRAF-6 and their downstream p-JNK/p-NF-κB/NLRP3/IL-1β-IL-18 signals in the colon (**Figure S15**). Therefore, in the present study, CRAMP may inhibit the activation of colonic MyD88/JNK/NF-κB/NLRP3 signaling pathway, subsequently suppressing the recruitment of IFN-γ^+^ T cells and their migration to the pancreas in* C. rodentium*-accelerated diabetes.

Our present study reports a role of CRAMP in gut-pancreas crosstalk to modify the recruitment of intestinal IFN-γ^+^ T cells and their translocation to the pancreas, subsequently regulating T1D development. The rising incidence of T1D and other pancreatic diseases observed in western countries may be associated with intestinal infections. Therefore, exogenous cathelicidins supplementation or manipulation of cathelicidins *via* a specific diet to restore a healthy intestinal barrier may represent an attractive therapeutic strategy to maintain intestinal homeostasis and protect against T1D.

## Supplementary Material

Supplementary figures and tables.

## Abbreviations

APCs: antigen-presenting cells
AMPs: antimicrobial peptides
ANOVA: one-way analysis of variance
CRAMP: cathelicidin-related antimicrobial peptide
*Cnlp*^-/-^ mice: CRAMP-deficient mice
*C. rodentium*: *Citrobacter rodentium*
DCs: dendritic cells
ELISA: enzyme-linked immunosorbent assays kit
GALTs: gut-associated lymphoid tissues
GFP^+^ *L. plantarum*: *Lactobacillus plantarum* with green fluorescent protein labeled plasmid
GC-MS: gas chromatography coupled mass spectrometry
H&E: hematoxylin-eosin staining
HRP-conjugated secondary antibodies: fluorescently labeled horseradish peroxidase-conjugated secondary antibodies
IFN-γ: interferon-gamma
MΦ: macrophages
M1 MΦ: inflammatory macrophages
M2 MΦ: regulatory macrophages
MLNs: mesenteric lymph nodes
NOD mice: non-obese diabetic mice
PBS: phosphate buffer saline
PCoA: principal component analysis
PLNs: pancreatic lymph nodes
SCFAs: short-chain fatty acids
SEM: standard error of mean
STZ: streptozotocin
T1D: type 1 diabetes
Tc1 cells: cytotoxic T cells type 1 cells
Th1 cells: T helper type 1 cells
TJPs: tight junction proteins
Tregs: foxp3^+^ regulatory T cells
WT mice: wild-type mice
