# Supplementary material for: Cathelicidin-related antimicrobial peptide protects against enteric pathogen-accelerated type 1 diabetes in mice
Source: Theranostics. 2022 Apr 24;12(7):3438–55. doi: 10.7150/thno.61433 (PMC9065195; doi:10.7150/thno.61433)
Supplement: Supplementary file 1 — Supplementary figures and tables. [file thnov12p3438s1.pdf]

Supplementary Material

Table S1-Information of the antibody for FACS

Antibody	Brand	Fluorescence	cat. # and recommended usage
CD86	BioLegend	Alexa Fluor 488	105018; 2 μg/test
CD206	BioLegend	Alexa Fluor 488	141710; 1 μg/test
F4/80	BioLegend	Brilliant Violet 421	123132; 5 μl/test
CD45	BioLegend	APC/Cy7	103154; 0.25 μg/test
CD11b	BioLegend	APC	101212; 0.25 μg/test
CD11c	BioLegend	PE	117308; 0.25 μg/test
CD4	Miltenyi	PE-Vio770	130-109-416; 5 μl/test
IFN-γ	BioLegend	APC	505810; 1 μg/test
CD8a	BioLegend	Alexa Fluor 488	100723; 0.25 μg/test
Foxp3	eBioscience	APC	17-5773-82; 1 μg/test
α4β7	BioLegend	PE	120606; 1 μg/test

Table S2-Information of the antibody for MΦ and conventional DCs in diabetes-prone NOD mice by FACS

Antibody	Brand	Fluorescence	#Lot and recommended usage
CD86	BioLegend	APC	105011; 1.25 μl/test
CD206	BioLegend	PE	141705; 2.5 μl/test
F4/80	BioLegend	Brilliant Violet 421	123137; 1.25 μl/test
CD45	BioLegend	PE-Cy7	103114; 1.25 μl/test
CD11b	BioLegend	Brilliant Violet 605™	101257; 1.25 μl/test
CD11c	BioLegend	Brilliant Violet 711™	117349; 0.3 μl/test

Table S3-Primer sequences used in this study for butyrate-producing bacteria abundance and CRAMP

Gene name	Gene symbol	Sequence
butyrate kinase gene	*buk*	Buk-5F1 CCATGCATTAAATCAAAAAGC
		Buk-5F2 CCATGCGTTAAACCAAAAAGC
		Buk-6R1 AGTACCTCCACCCATGTG
		Buk-6R2 AATACCTCCGCCCATATG
		Buk-6R3 AATACCGCCRCCCATATG
Total bacteria	Total	Forward 5’- GCAGGCCTAACACATGCAAGTC
		Reverse 5’-CTGCTGCCTCCCGTAGGAGT
butyryl-coenzym	*but-CoA*	Forward5’-GCIGAICATTTCACITGGAAYWSITGGCAYATG
		Reverse5’-CCTGCCTTTGCAATRTCIACRAANGC
β-actin	*β-actin*	Forward5’-CCCAGGCATTGCTGACAGG-3’
		Reverse5’-TGGAAGGTGGACAGTGAGGC-3’
CRAMP	*CRAMP*	Forward5’-CTTCAAGGAACAGGGGGTGG-3’
		Reverse5’-CTTGAACCGAAAGGGCTGTG-3’

Table S4-Information of the antibody for Western blot

Antibody	Brand	cat. # and dilution ratio
p38 MAPK	CST	9212S; 1:1000
p-ERK1/2	CST	4370S; 1:1000
p65 (Ser536)	CST	3033S; 1:1000
p-P38 MAPK	CST	9211S; 1:1000
p-JNK (Thr183/Tyr185)	CST	9251S; 1:1000
SAPK/JNK	CST	9252S; 1:1000
Erk1/2	CST	4695S; 1:1000
TRAF6	Abcam	ab40675; 1:5000
ZO-1	Invitrogen	33-9100; 2μg/ml
ZO-2	Invitrogen	38-9100; 2μg/ml
Occludin	Santa Cruz	sc-8144; 1:200
CRAMP	innovagen	PA-CRPL-100
β-actin	CST	3700T; 1:1000
MyD88	CST	4283S; 1:1000
IRAK-4	CST	4363S; 1:1000
p-NFκB p65 (Ser 536)	Santa Cruz	sc-136548; 1:500
NLRP3 (D4D8T)	CST	15101S; 1:1000
Cleaved-IL-1β	CST	52718S; 1:1000
Cleaved-IL-18	Abcam	ab71495; 1 μg/ml
Caspase-1-p20	Santa Cruz	398715; 1:1000

Table S5-Information for the main instrument

Instrument	Manufacturer and/or address
Individual ventilated caging systems	TECNIPLAST, Italy
Glucometer glucometer	Roche, NSW, USA
Invitrogen™ Attune™ NxT Flow Cytometer	Thermo Fisher Scientific, Massachusetts, USA
Digital slice scanner	3DHISTCH, Hungary
Western Lightening Plus-ECL	Pierce, Rockford, IL, USA
GC and coupled to the MS detector (GCMS-QP2010)	Shimadzu, Japan
Rtx-WAX capillary column (30 m×0.25 mm×0.25 μm)	Bellefonte, PA, USA
Gentle MACS™ Dissociators	MiltenyiBiotec, BergischGladbach, Germany
Illumina MiSeq	Illumina
High-throughput tissue burnisher	SCIENTZ-48, Ningbo, China

Table S6-Information for the main reagent

Reagent	Manufacturer and/or address
Pentobarbital sodium	Sigma-Aldrich, Saint Louis, MO, USA
Protease inhibitors	Beyotime, Shanghai, China
BCA protein assay Kit	Beyotime, Shanghai, China
C. rodentium strain DBS100	ATCC 51459; American Type Culture Collection
Streptozotocin	Sigma S0130, St. Louis, MO, USA
Cell stimulation cocktail	eBioscience, San Diego, CA, USA
Trizol reagent	Invitrogen
Fast-Start SYBR Green PCR reagents	Roche
ELISA kit for CRAMP	CUSABIO BIOTECH CO., LTD, Wuhan, China
Fast DNA Spin Kit for Soil	MP Biomedicals, cat. # 6560-200, California, USA
Gene Clean Turbo	MP Biomedicals, cat. # 111102400
Quant-iTPicoGreen dsDNA Assay Kit	Life Technologies, cat. # P7589
TruSeq DNA LT Sample Preparation Kit	Illumina, cat. # FC-121-2001, San Diego, USA
MiSeqReagent Kit	500 cycles-PE, cat. # MS-102-2003

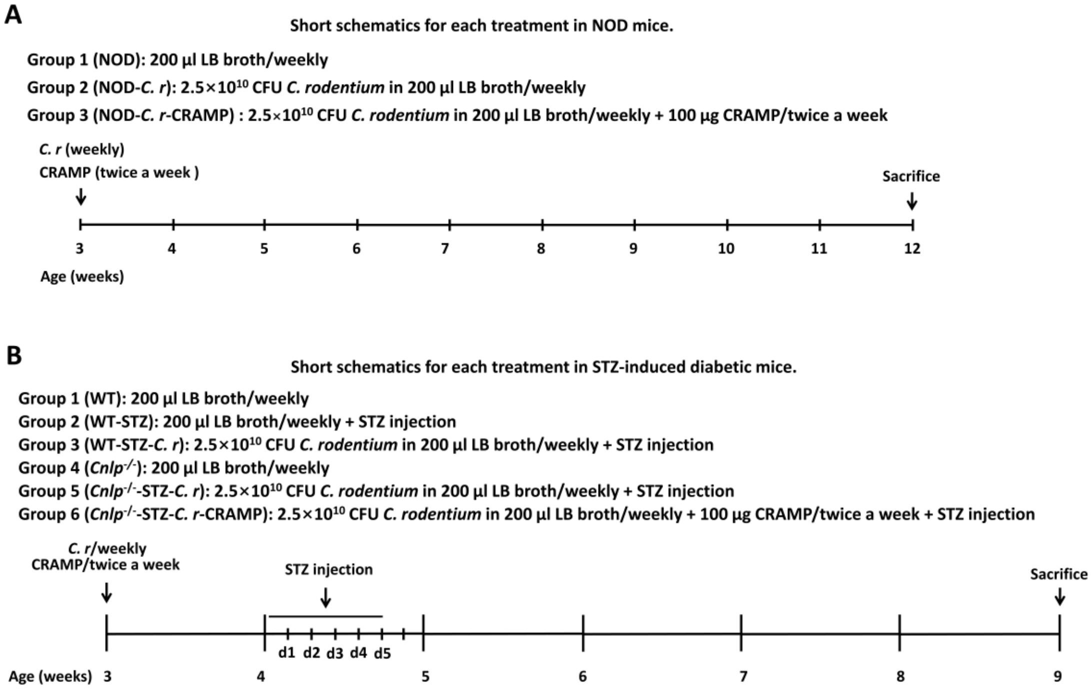

Figure S1. The short schematics for each treatment. (**A**) To evaluate the role and gut-pancreas pathophysiological mechanism of CRAMP in enteric pathogen-accelerated T1D, NOD mice were randomly assigned to three groups to receive CRAMP (2×100 μg) or equal volumes of saline by intraperitoneal injection twice a week after *C. rodentium* infection (2.5×10^{10} CFU in 200 μL LB broth) from 3 to 12 weeks of age. (**B**) Three-week-old WT and *Cnlp*$^{-/-}$ mice were infected with the *C. rodentium* (2.5×10^{10} CFU in 200 μL LB broth) weekly along with the corresponding intervention as previously described from 3 to 9 weeks of age (n = 12). Infected mice were then injected with low-dose (45 mg·kg^{-1} a day) of STZ by intraperitoneal injection for 5 consecutive days to induce T1D. All the mice received two injections of CRAMP (2×100 μg) or saline by intraperitoneal injection twice a week. Mice were monitored for the glucose from the tail vein after 6 h fasting and euthanized at the end of the experiment for immune cell detection by flow cytometry.

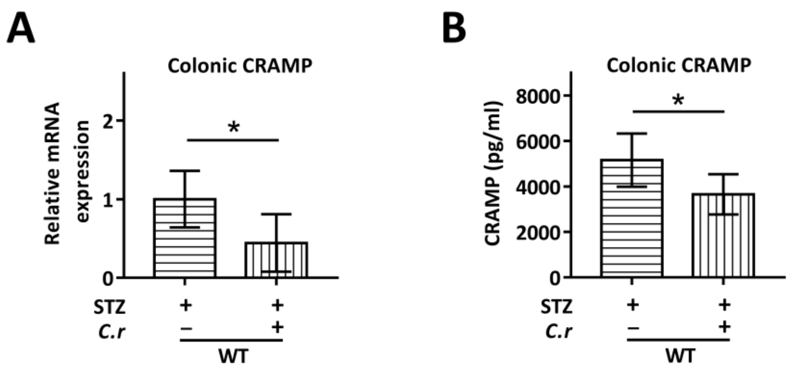

Figure S2. Colonic CRAMP production is defective in *C. rodentium*-accelerated T1D. Colonic CRAMP expression determined by real-time PCR (**A**), and ELISA (**B**). Data are mean ± SEM (n = 8-12). * $p < 0.05$.

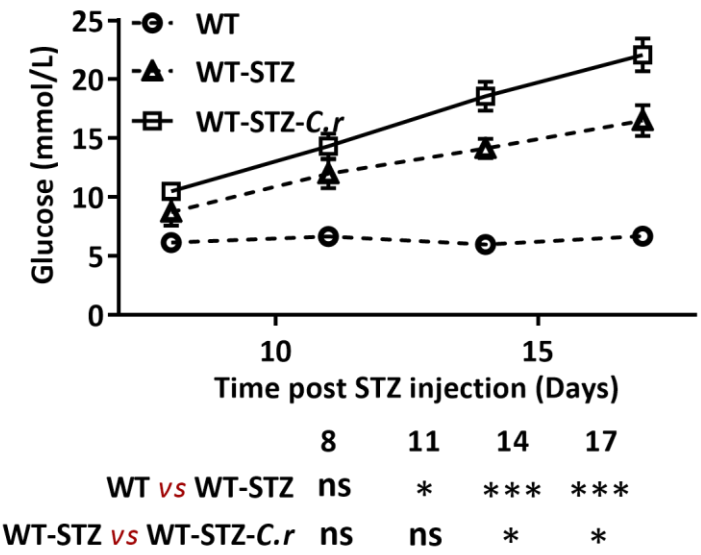

Figure S3. *C. rodentium* infection correlates with higher fasting glucose. Blood glucose was determined after 6 h fasting in STZ-induced diabetic mice. Data are mean ± SEM (n = 4-8). * $p < 0.05$, ** $p < 0.01$, *** $p < 0.001$.
WT-STZ mice: the WT mice treated with STZ; **WT-STZ-*C.r* mice**: the *C. rodentium*-infected WT mice treated with STZ.

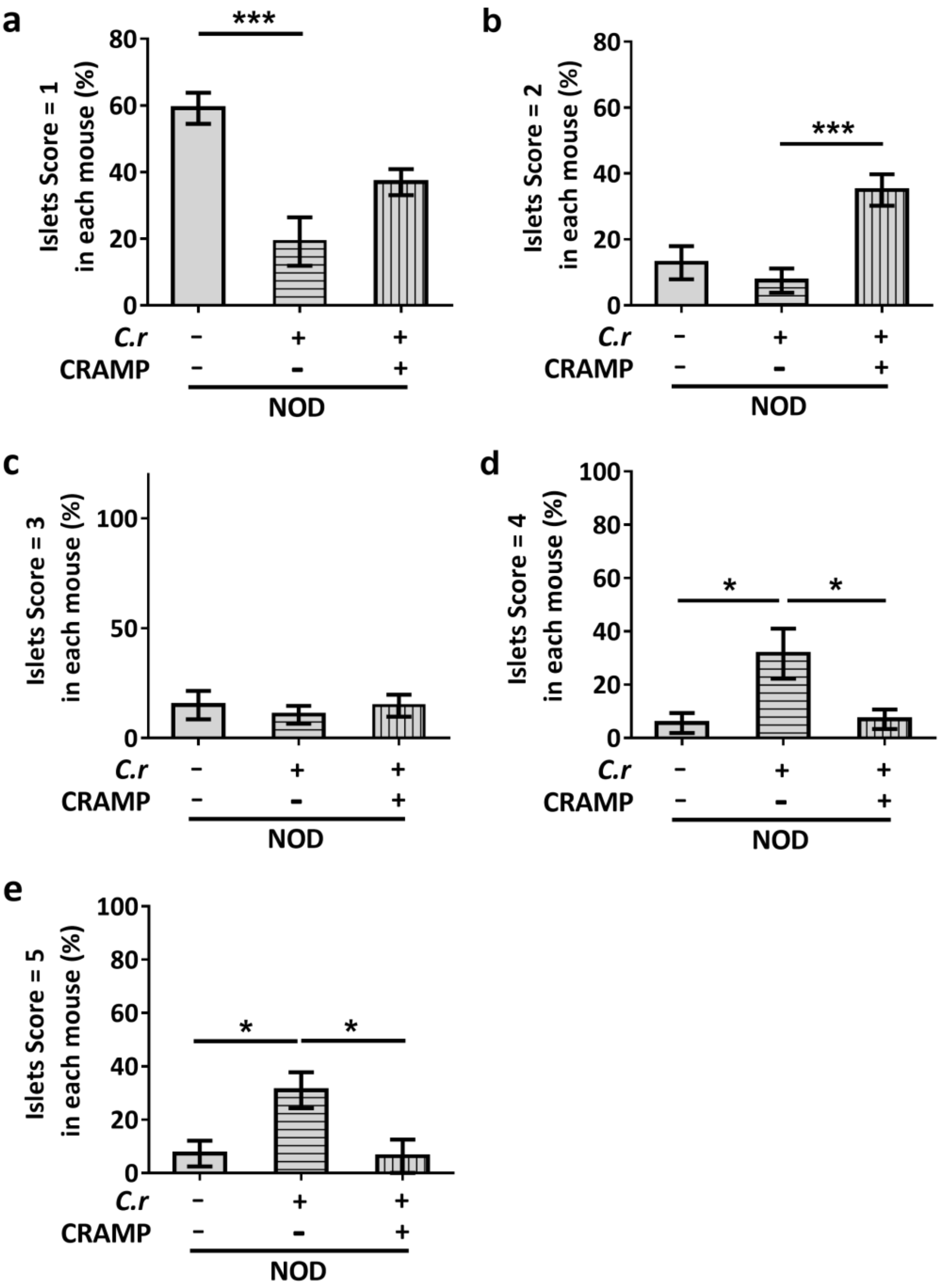

Figure S4. Insulitis and insulitis score in NOD mice at 12 weeks of age. 1 = white, no infiltration; 2 = light gray, few mononuclear cells infiltrated; 3 = gray, peri-insulitis; 4 = dark gray, < 50% islet infiltration; 5 = black, > 50 % islet infiltration. Data are mean ± SEM (n = 8). * $p < 0.05$, *** $p < 0.001$.

NOD mice: the NOD control mice; **NOD-*C.r* mice**: the *C. rodentium*-infected NOD mice; **NOD-*C.r*-CRAMP mice**: the *C. rodentium*-infected NOD mice treated with CRAMP.

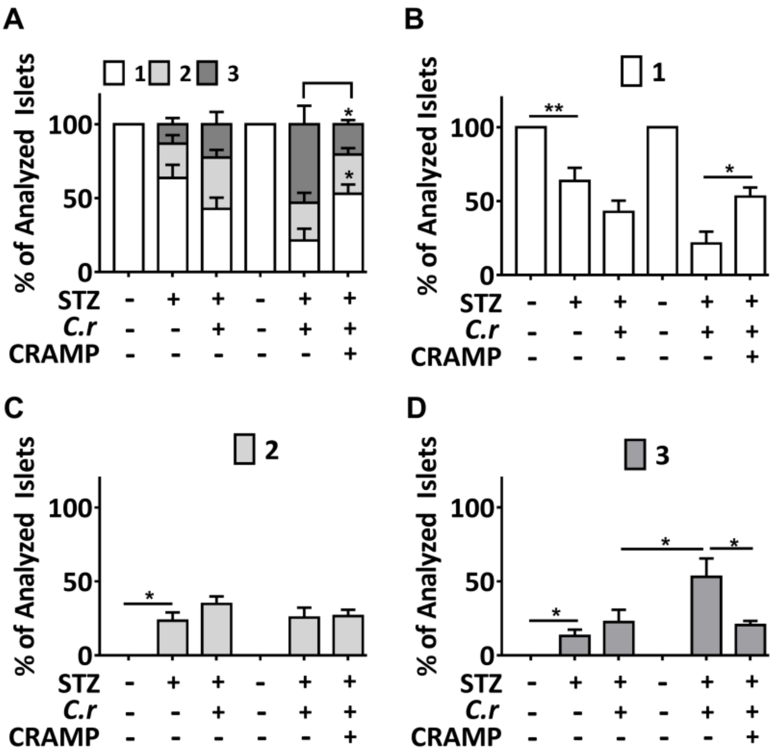

Figure S5. Insulitis and insulitis score in STZ-induced diabetic mice. 1 = white, no infiltration; 2 = light gray, mild inconsistent pancreatic islet cell size, islet atrophic, hyperplasia around the pancreatic duct and pancreatic duct expansion; 3 = gray, severe inconsistent pancreatic islet cell size, islet atrophic, hyperplasia around the pancreatic duct and pancreatic duct expansion. Data are mean ± SEM (n = 6). * $p < 0.05$, ** $p < 0.01$.

WT mice: WT control mice; **WT-STZ mice**: the WT mice treated with STZ; **WT-STZ-*C.r* mice**: the *C. rodentium*-infected WT mice treated with STZ; ***Cnlp***$^{-/-}$**-STZ-*C.r* mice**: the *C. rodentium*-infected *Cnlp*$^{-/-}$ mice treated with STZ; ***Cnlp***$^{-/-}$**-STZ-*C.r*-CRAMP mice**: the *C. rodentium*-infected *Cnlp*$^{-/-}$ mice treated with STZ and CRAMP.

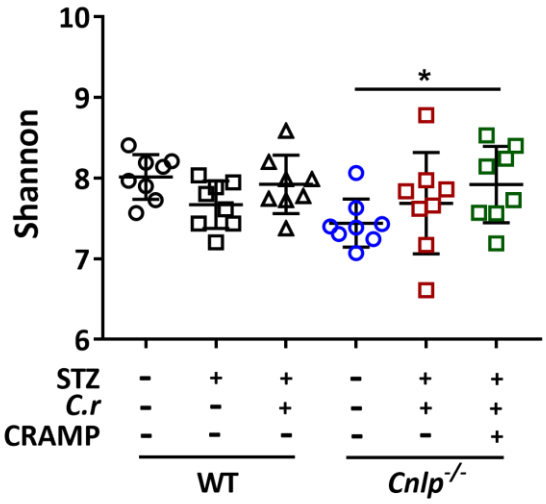

Figure S6. The Shannon indexes (diversity index). Data are mean ± SEM (n = 8). * $p < 0.05$.

WT mice: WT control mice; **WT-STZ mice**: the WT mice treated with STZ; **WT-STZ-*C.r* mice**: the *C. rodentium*-infected WT mice treated with STZ; ***Cnlp*$^{-/-}$ mice**: the *Cnlp*$^{-/-}$ control mice; ***Cnlp*$^{-/-}$-STZ-*C.r* mice**: the *C. rodentium*-infected *Cnlp*$^{-/-}$ mice treated with STZ; ***Cnlp*$^{-/-}$-STZ-*C.r*-CRAMP mice**: the *C. rodentium*-infected *Cnlp*$^{-/-}$ mice treated with STZ and CRAMP.

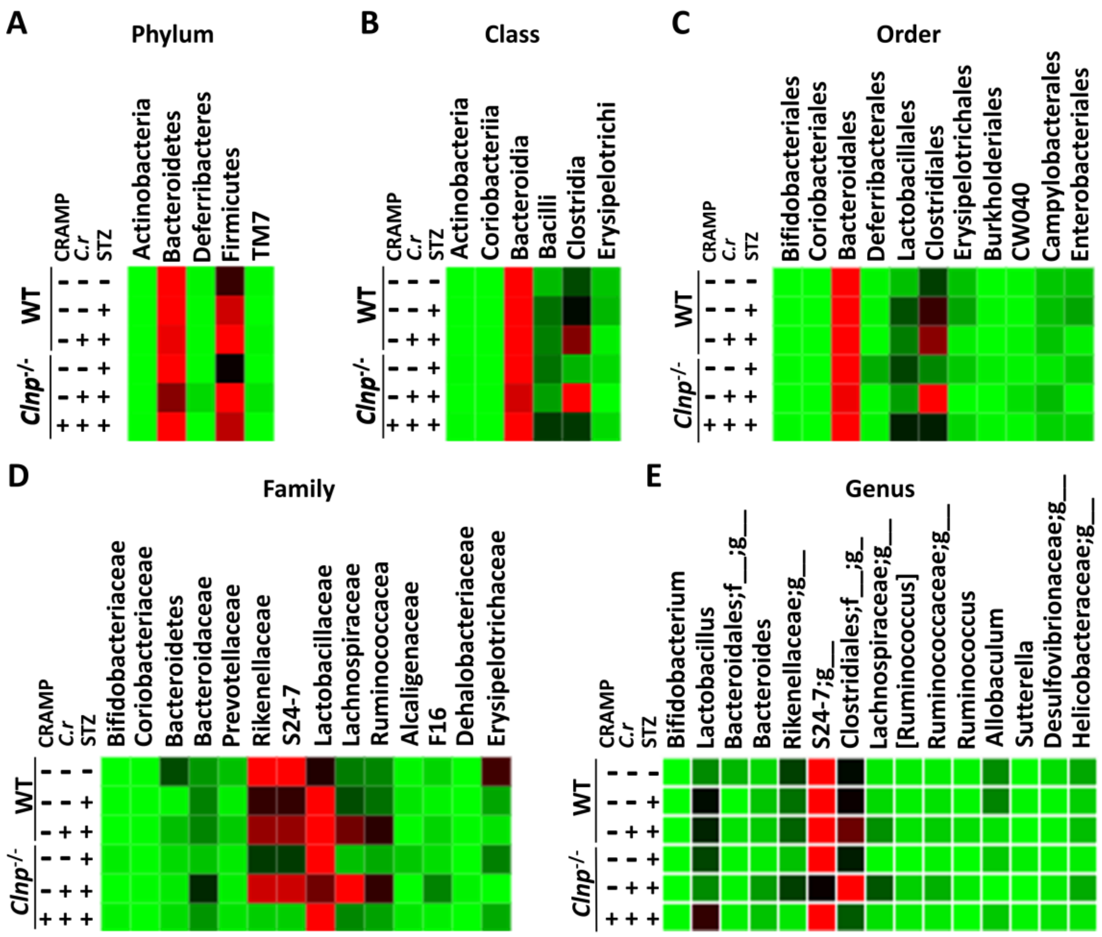

Figure S7. The relative abundance of the main altered phyla, classes, orders, families, and genera were shown by heat map. The relative abundance of the main altered phyla (**A**), classes (**B**), orders (**C**), families (**D**), and genera (**E**) in each group. Data are mean ± SEM (n = 8).

WT mice: WT control mice; **WT-STZ mice**: the WT mice treated with STZ; **WT-STZ-*C.r* mice**: the *C. rodentium*-infected WT mice treated with STZ; ***Cnlp*$^{-/-}$ mice**: the *Cnlp*$^{-/-}$ control mice; ***Cnlp*$^{-/-}$-STZ-*C.r* mice**: the *C. rodentium*-infected *Cnlp*$^{-/-}$ mice treated with STZ; ***Cnlp*$^{-/-}$-STZ-*C.r*-CRAMP mice**: the *C. rodentium*-infected *Cnlp*$^{-/-}$ mice treated with STZ and CRAMP.

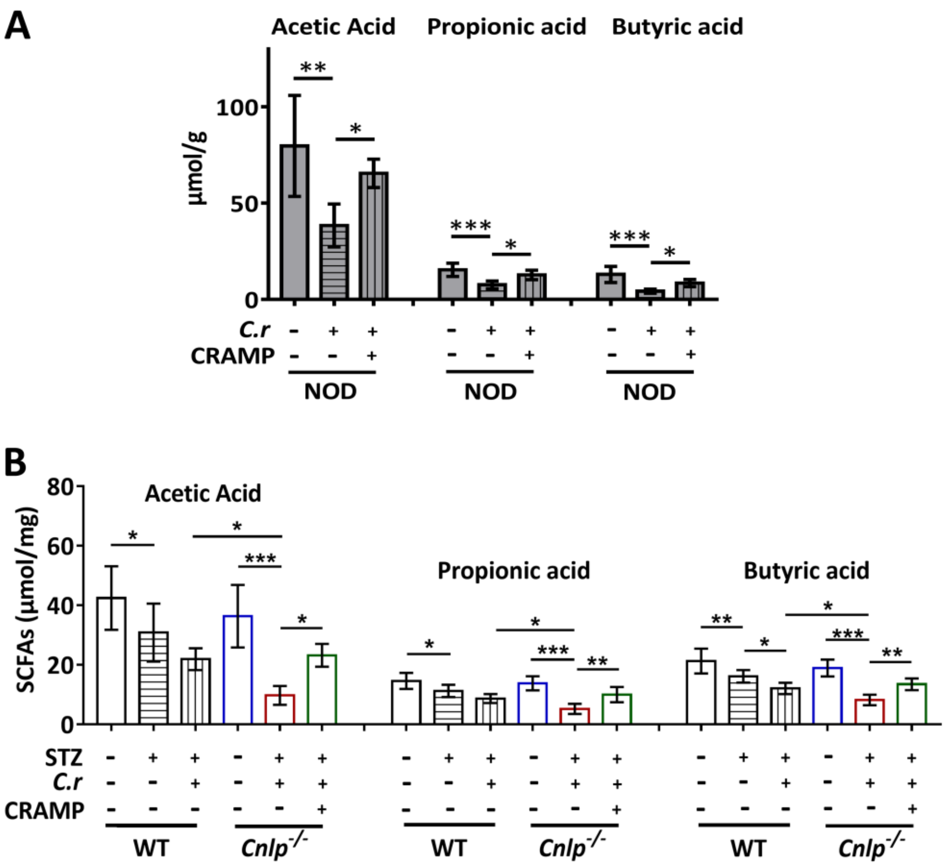

Figure S8. Fecal SCFAs concentration in the NOD (*A*) and STZ-induced diabetic (*B*) mice measured by GC-MS. Data are mean ± SEM (n = 8). * $p < 0.05$, ** $p < 0.01$, *** $p < 0.001$.

NOD mice: the NOD control mice; **NOD-*C.r* mice**: the *C. rodentium*-infected NOD mice; **NOD-*C.r*-CRAMP mice**: the *C. rodentium*-infected NOD mice treated with CRAMP; **WT mice**: WT control mice; **WT-STZ mice**: the WT mice treated with STZ; **WT-STZ-*C.r* mice**: the *C. rodentium*-infected WT mice treated with STZ; ***Cnlp*$^{-/-}$ mice**: the *Cnlp*$^{-/-}$ control mice; ***Cnlp*$^{-/-}$-STZ-*C.r* mice**: the *C. rodentium*-infected *Cnlp*$^{-/-}$ mice treated with STZ; ***Cnlp*$^{-/-}$-STZ-*C.r*-CRAMP mice**: the *C. rodentium*-infected *Cnlp*$^{-/-}$ mice treated with STZ and CRAMP.

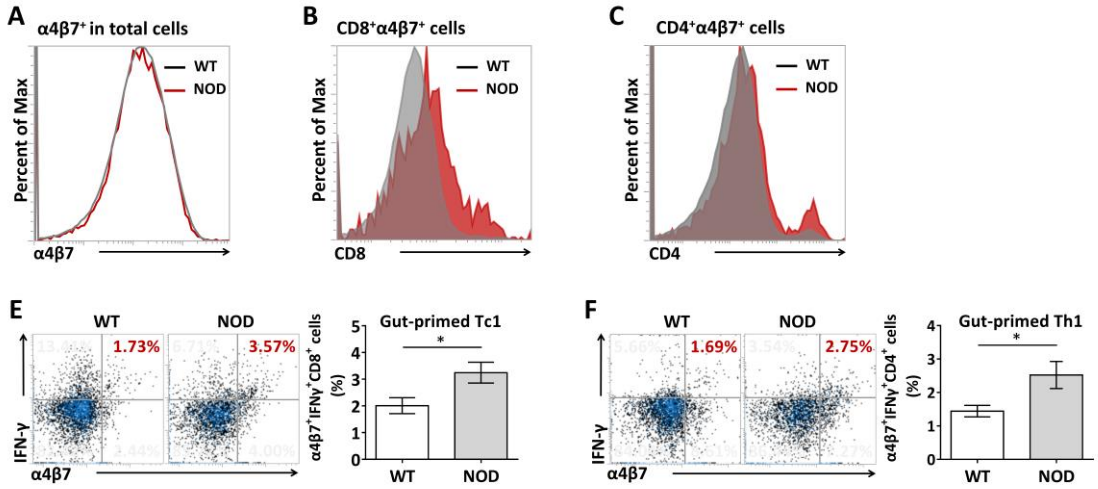

Figure S9. Diabetes-prone NOD mice harbored more autoreactive α4β7$^+$ cells and effector T cells in the pancreas than the healthy WT mice. (**A**) Percent of α4β7$^+$ cells in total pancreatic cells (gut-primed cells) in WT and NOD mice. (**B**) Percent of α4β7$^+$CD8$^+$ cells in α4β7$^+$ cells (gut-primed CD8$^+$ T-cells) in the pancreas of WT and NOD mice. (**C**) Percent of α4β7$^+$CD4$^+$ cells in α4β7$^+$ cells (gut-primed CD4$^+$ T-cells) in the pancreas of WT and NOD mice. (**E**) Percent of α4β7$^+$CD8$^+$IFN-γ$^+$ cells in CD8$^+$ cells (gut-primed Tc1) from the pancreas of WT and NOD mice. (**F**) Percent of α4β7$^+$CD4$^+$IFN-γ$^+$ cells in CD4$^+$ cells (gut-primed Th1 cells) from the pancreas of WT and NOD mice. Data are mean ± SEM (n = 4-5). *t*-test was used for two independent groups. * $p < 0.05$.

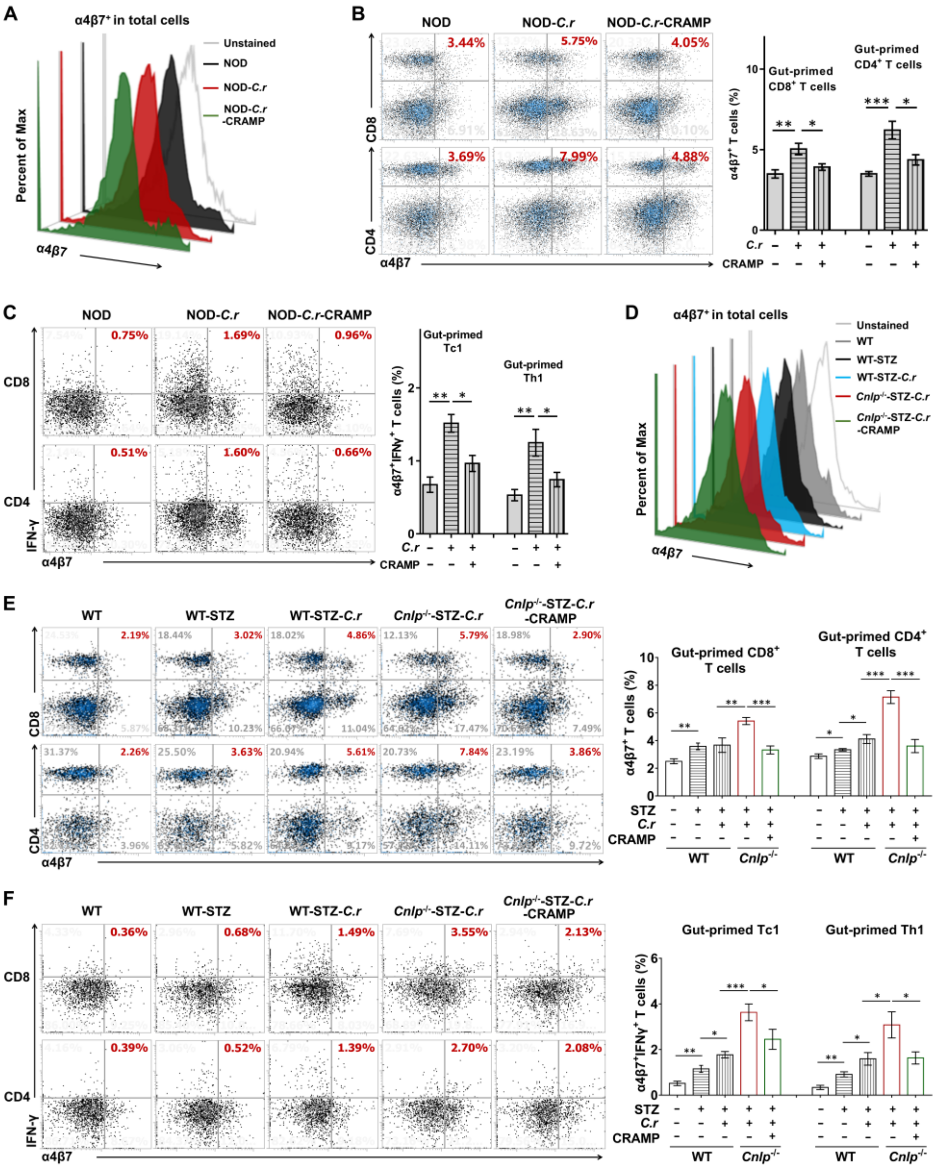

Figure S10. CRAMP inhibits the migration of gut-primed IFN-γ^+ T cells to the PLNs in *C. rodentium*-accelerated T1D. (**A**) The percent of $\alpha4\beta7^+$ cells in total PLNs cells (gut-primed cells) from NOD mice. (**B**) The percent of $\alpha4\beta7^+CD8^+$ cells in $\alpha4\beta7^+$ cells (gut-primed $CD8^+$ T cells) in the PLNs from NOD mice; the percent of $\alpha4\beta7^+CD4^+$ cells in $\alpha4\beta7^+$ cells (gut-primed $CD4^+$ T cells) from the PLNs of NOD mice. (**C**) The percent of $\alpha4\beta7^+CD8^+$IFN-γ^+ cells in $CD8^+$ cells (gut-primed Tc1) from the PLNs of NOD mice; the percent of $\alpha4\beta7^+CD4^+$IFN-γ^+ cells in $CD4^+$ cells (gut-primed Th1) from the PLNs of NOD mice. (**D**) The percent of $\alpha4\beta7^+$ cells in total PLNs cells (gut-primed cells) from STZ-induced diabetic mice. (**E**) The percent of $\alpha4\beta7^+CD8^+$ cells in $\alpha4\beta7^+$ cells (gut-primed $CD8^+$ T cells) in the PLNs of STZ-induced diabetic mice; the percent of $\alpha4\beta7^+CD4^+$ cells in $\alpha4\beta7^+$ cells (gut-primed $CD4^+$ T cells) from the PLNs of STZ-induced diabetic mice. (**F**) The percent of $\alpha4\beta7^+CD8^+$IFN-γ^+ cells in $CD8^+$ cells (gut-primed Tc1) from the PLNs of STZ-induced diabetic mice; the percent of $\alpha4\beta7^+CD4^+$IFN-γ^+ cells in $CD4^+$ cells (gut-primed Th1 cells) from the PLNs of STZ-induced diabetic mice. Data are mean ± SEM. n = 5-6. * $p < 0.05$, ** $p < 0.01$, *** $p < 0.001$.

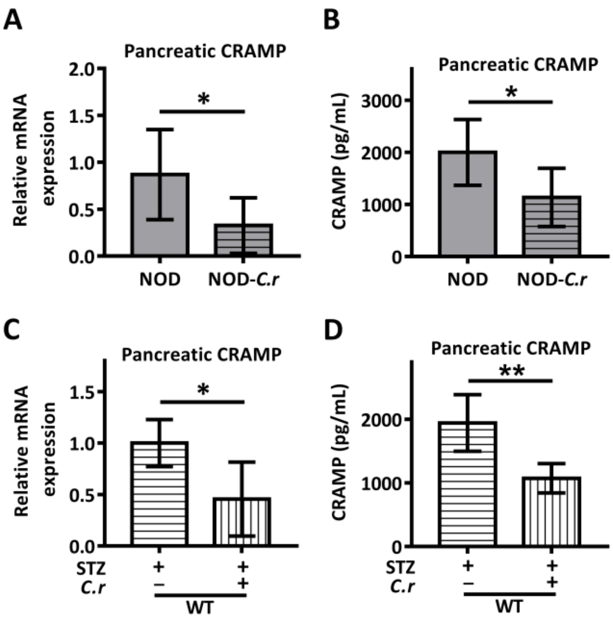

Figure S11. Pancreatic CRAMP production is defective in *C. rodentium*-accelerated T1D. Pancreatic CRAMP was determined by real-time PCR (**A**) and ELISA (**B**) in NOD mice. Pancreatic CRAMP was determined by real-time PCR (**C**) and ELISA (**D**) in STZ-induced diabetic mice. Data are mean ± SEM (n = 4-6). * $p < 0.05$, ** $p < 0.01$. **NOD mice**: the NOD control mice; **NOD-*C.r* mice**: the *C. rodentium*-infected NOD mice.

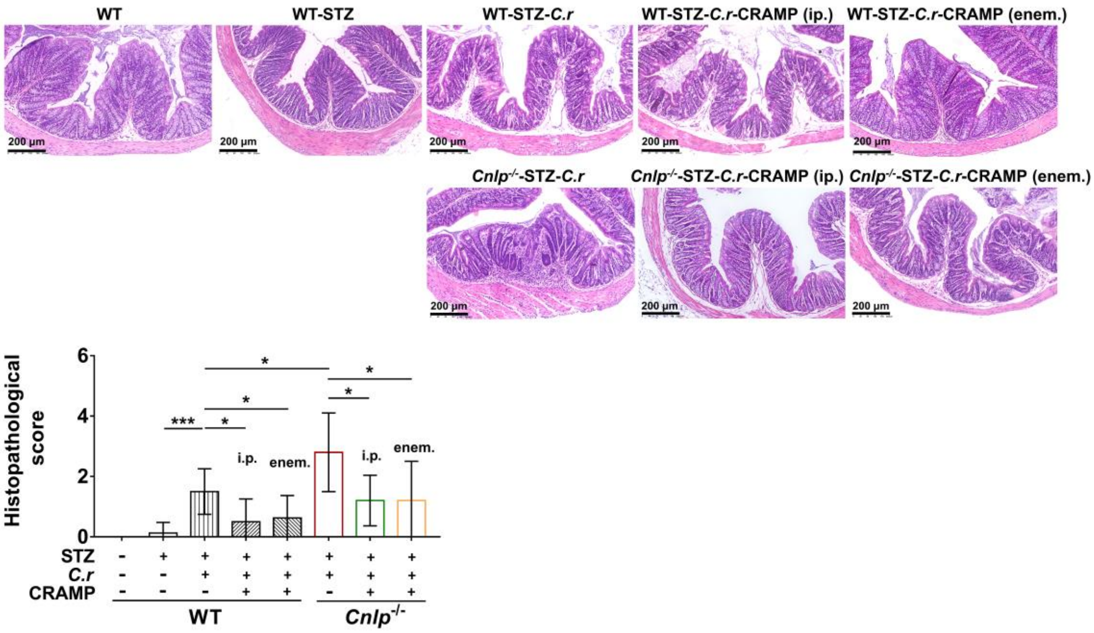

Figure S12. CRAMP by intracolonic administration attenuates colonic barrier disruption in *C. rodentium*-accelerated T1D. H&E staining of the colon and histopathological score in STZ-induced diabetic mice. Scale bar: 200 µm. Data are mean ± SEM, n = 5-8. * $p < 0.05$, ** $p < 0.01$, *** $p < 0.001$.

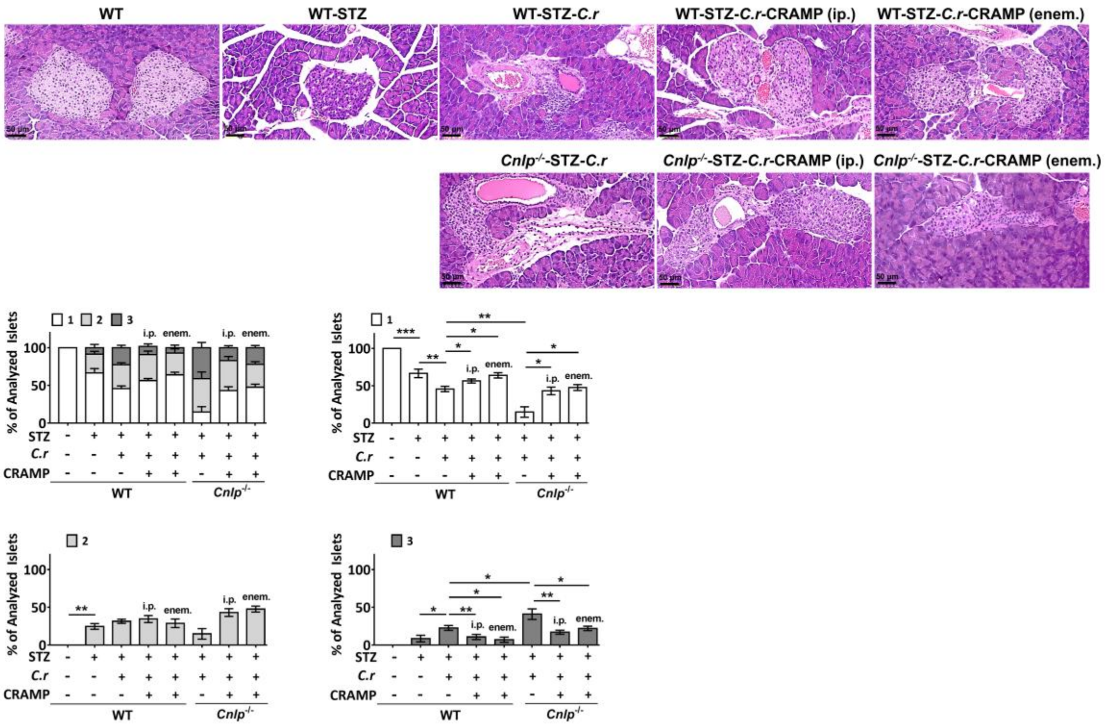

Figure S13. CRAMP by intracolonic administration protects against *C. rodentium*-accelerated insulitis. Insulitis and insulitis score in STZ-induced diabetic mice. 1 = white, no infiltration; 2 = light gray, mild inconsistent pancreatic islet cell size, islet atrophic, hyperplasia around the pancreatic duct and pancreatic duct expansion; 3 = gray, severe inconsistent pancreatic islet cell size, islet atrophic, hyperplasia around the pancreatic duct and pancreatic duct expansion. n = 5-6. Scale bar: 50 μm. Data are mean ± SEM. * $p < 0.05$, ** $p < 0.01$, *** $p < 0.001$.

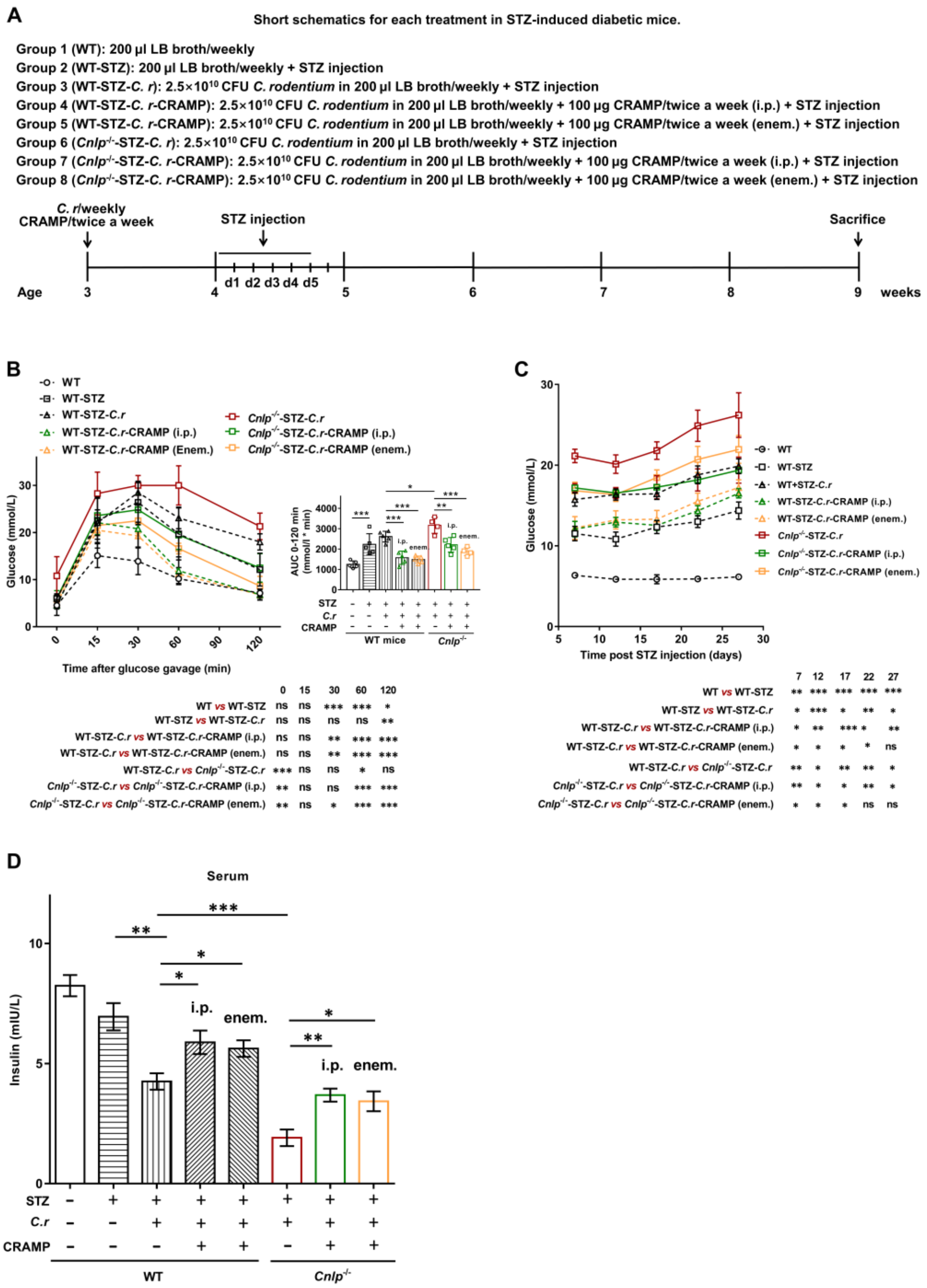

Figure S14. CRAMP by intracolonic administration protects against *C. rodentium*-accelerated STZ-induced diabetes. (**A**) Animal protocol. Three-week-old WT and *Cnlp*$^{-/-}$ mice were infected with the *C. rodentium* weekly along with the corresponding intervention as previously described from 3 to 9 weeks of age. Infected mice were then injected with low-dose of STZ by intraperitoneal injection for 5 consecutive days to induce T1D. All the mice received CRAMP (100 μg, twice a week) or saline by intraperitoneal injection or by intracolonic administration. Mice were monitored for the glucose from the tail vein after 6 h fasting. (**B**) Glucose tolerance tests were performed after 18 h fasting at 3 weeks after STZ injection, n = 4-5. (**C**) Blood glucose was tested after 6 h fasting, n = 5-8. (**D**) Serum insulin levels, n = 5-8. Data are mean ± SEM. * $p < 0.05$, ** $p < 0.01$, *** $p < 0.001$.

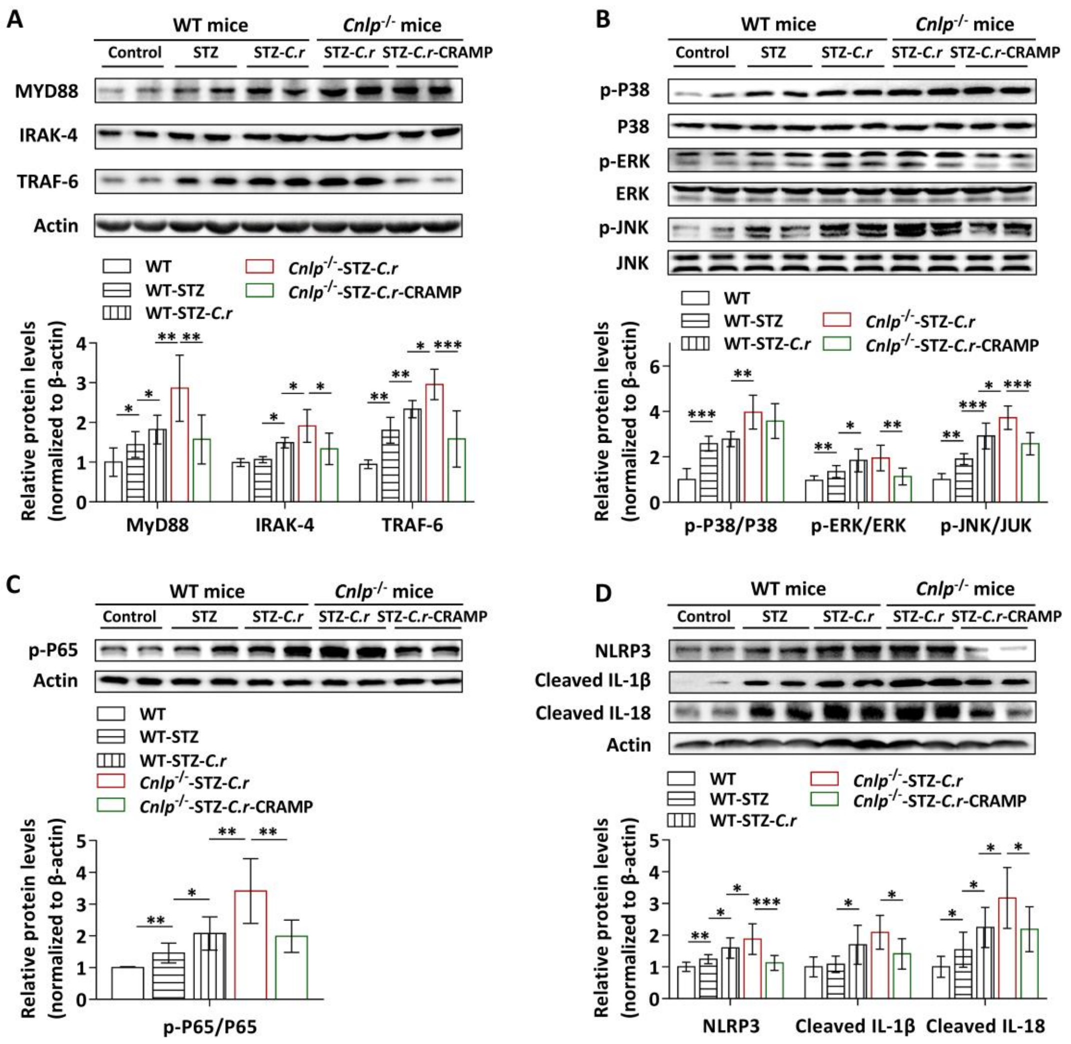

Figure S15. CRAMP inhibits activation of colonic MyD88-NLRP3 signaling pathway in *C. rodentium*-accelerated diabetes. (**A**) MyD88, IRAK-4, TRAF6 determined by Western blot, and the grey value analysis by Image J. (**B**) MAPKs pathway determined by Western blot, and the grey value analysis by Image J. (**C**) NF-κB determined by Western blot, and the grey value analysis by Image J. (**D**) NLRP3, IL-1β and IL-18 determined by Western blot, and the grey value analysis by Image J. Data are mean ± SEM, n = 8. * $p < 0.05$, ** $p < 0.01$, *** $p < 0.001$.
